# Integrated transcriptomic and pathway analyses of sorghum plants revealed the molecular mechanisms of host defense against aphids

**DOI:** 10.3389/fpls.2024.1324085

**Published:** 2024-06-06

**Authors:** Kumar Shrestha, Jian Huang, Liuling Yan, Andrew N. Doust, Yinghua Huang

**Affiliations:** ^1^ Department of Plant Biology, Ecology and Evolution, Oklahoma State University, Stillwater, OK, United States; ^2^ Department of Plant and Soil Sciences, Oklahoma State University, Stillwater, OK, United States; ^3^ Plant Science Research Laboratory, United States Department of Agriculture - Agricultural Research Service (USDA-ARS), Stillwater, OK, United States

**Keywords:** biotic stress, defense pathways, phytohormones, plant resistance, plant-insect interaction, sorghum, sugarcane aphid, transcriptomics

## Abstract

Sugarcane aphid has emerged as a major pest of sorghum recently, and a few sorghum accessions were identified for resistance to this aphid so far. However, the molecular and genetic mechanisms underlying this resistance are still unclear. To understand these mechanisms, transcriptomics was conducted in resistant Tx2783 and susceptible BTx623 sorghum genotypes infested with sugarcane aphids. A principal component analysis revealed differences in the transcriptomic profiles of the two genotypes. The pathway analysis of the differentially expressed genes (DEGs) indicated the upregulation of a set of genes related to signal perception (nucleotide-binding, leucine-rich repeat proteins), signal transduction [mitogen-activated protein kinases signaling, salicylic acid (SA), and jasmonic acid (JA)], and plant defense (transcription factors, flavonoids, and terpenoids). The upregulation of the selected DEGs was verified by real-time quantitative PCR data analysis, performed on the resistant and susceptible genotypes. A phytohormone bioassay experiment showed a decrease in aphid population, plant mortality, and damage in the susceptible genotype when treated with JA and SA. Together, the results indicate that the set of genes, pathways, and defense compounds is involved in host plant resistance to aphids. These findings shed light on the specific role of each DEG, thus advancing our understanding of the genetic and molecular mechanisms of host plant resistance to aphids.

## 1 Introduction

Sorghum [*Sorghum bicolor* (L.) Moench] is an important cereal crop in the world for food, feed, and biofuels. It ranks fifth in terms of both production and area planted among cereal crops, yet sorghum production declined in 2015/2016 and 2016/2017 by 21.6% and 19.5%, respectively (USDA, 2019). The loss in production is attributed to abiotic and biotic stresses, of which sugarcane aphid (*Melanaphis sacchari* Zehntner) infestation is currently one of the important causes. A major outbreak of sugarcane aphid in sorghum occurred in Texas (2013), before spreading to more than 20 states in the USA. It is now considered as a major pest of sorghum (Bowling et al., 2016). The sugarcane aphid, a phloem-feeding insect, can attack sorghum at all developmental stages, resulting in severe damage to sorghum plants and significant yield loss (Singh et al., 2004). While feeding, the aphid pierces the phloem with its stylet and sucks the plant nutrients. In addition, it produces honeydew that ultimately reduces the photosynthetic area, affects the seed set, and hinders the harvesting process (Fiehn, 2002; Carena and Glogoza, 2004). Furthermore, the aphid can cause indirect damages by transmitting sugarcane yellow leaf virus among plants, which can result in 20%–40% yield losses in sugarcane cultivars (Rassaby et al., 2003).

Host plant resistance (HPR) is an essential tool for pest management as it is effective, economical, and environmentally friendly. Several sorghum genotypes have a high level of resistance to sugarcane aphids, and it has been reported that the deployment of HPR was the most effective method to control the population of aphids when compared to insecticide treatment and planting date (Szczepaniec, 2018). The densities of aphids were 2–2.5 times higher in the susceptible genotypes in comparison with resistant ones (Kiani and Szczepaniec, 2018). The resistant genotypes provide resistance to pests through three main mechanisms: antibiosis, antixenosis, and tolerance. Antibiosis causes injury, death, reduced growth, reduced longevity, and fecundity of the pest. Antixenosis, also called non-preference, is based on host traits that deter insects from feeding. Plant tolerance enables the plant to remain healthy, maintaining growth and productivity when under attack by insects. Normally, resistant genotypes displaying more than one mechanism of resistance are considered better for cultivar development (Paudyal et al., 2019). However, the genetic and molecular mechanisms underlying resistant sorghum genotypes during sugarcane aphid infestation are not well understood.

Upon wounding by phloem-feeding insects like aphids, plants recognize elicitors and effectors of aphids and deploy both a constitutive defense and activate an induced defensive response that includes a broad change in gene expression and biochemical pathways (Thompson and Goggin, 2006; Hogenhout and Bos, 2011). The induced responses to aphid infestation by the resistant genotype activate diverse genes in a sequential flow starting from signal perception to signal transduction and ultimately defense response. After perception of elicitors from infestation, the effectors of aphids are recognized by R-proteins that are nucleotide-binding, leucine-rich repeat proteins (NLRs), and the host plant activates more specific effector-triggered immunity (ETI). The ETI triggers specific multifaceted resistance like hypersensitive response (HR), reactive oxygen species (ROS) accumulation, defense hormone synthesis, and mitogen-activated protein kinase (MAPK) signaling (Jones and Dangl, 2006; Dangl et al., 2013; Huang and Huang, 2023). Some of the NLR genes in plants that are used for defense against phloem feeding insects are *Mi-1.2* for aphid in tomato, *BPH9/14* for brown plant hopper (BPH) in rice, and *Vat* in melon that confers resistance to aphids, whitefly, and psyllid (Casteel et al., 2006; Du et al., 2009; Dogimont et al., 2014; Zhang et al., 2022).

After signal perception by R-proteins, signal transduction is conducted by calcium and MAPK signaling events, which, in turn, alters phytohormone biosynthesis, and reprograms the transcriptional activation of defense genes and accumulation of defensive metabolites (Hettenhausen et al., 2015). The phytohormone-related genes upregulated during aphid infestation are related to salicylic acid (SA), jasmonic acid (JA), and ethylene (ET) (Bari and Jones, 2009; Erb et al., 2012; Pieterse et al., 2012; Huang et al., 2022). Also, crosstalk between SA and JA signal transduction pathways is thought to fine-tune plant responses to infestation (Thaler et al., 2012). These signal transductions lead to synthesis of defense compounds like phenylpropanoids, flavonoids, terpenoids, and oxylipins, which are often toxic to insects and play key roles in defense (Bennett and Wallsgrove, 1994; Kessler and Baldwin, 2002). Over the last few years, transcriptomics studies have been popular as they not only can analyze global changes in gene expression but also can effectively identify the suite of defense genes and pathways activated during stress (Lowe et al., 2017). However, the genetic and molecular mechanisms of resistance in the sorghum-aphid interaction have not been well studied. Therefore, our research goal was to explore and understand the transcriptional responses of both resistant and susceptible sorghum genotypes to sugarcane aphid infestation at early and late infestation stages. We hypothesized that, during aphid infestation, expression of defense-related genes would be higher in the resistant sorghum genotype as compared to the susceptible one. An improved understanding of the molecular interactions between different genotypes (resistant and susceptible) with sugarcane aphids will provide insights into plant defense mechanisms and contribute better strategies in molecular breeding for effective crop protection.

## 2 Materials and methods

### 2.1 Sorghum plant growth and sugarcane aphid bioassay

Two sorghum genotypes, BTx623 (susceptible) and Tx2783 (resistant), were selected as parallel lines for this study. Sorghum seedlings were prepared in the greenhouse at constant temperature (28°C ± 2°C) and 60% relative humidity under the photoperiod of 14-h light/10-h dark. Sugarcane aphid colonies were cultured on the susceptible sorghum line Tx7000. Sorghum seedlings at the two- to three-leaf stage (8–10 days) were infested with 20 adult apterous sugarcane aphids to the adaxial surface of the first true leaf. Each of the infested and the control plants (not infested with aphids) were covered with a transparent cylindrical cage with nylon mesh on the top. To evaluate differential responses of the two genotypes to aphid infestation, changes in aphid number were recorded at 1, 3, 6, 9, and 12 days post infestation (dpi) from 10 independent plants of each infested line. In addition, plant damage scores were recorded using a scale of 0 to 6, with 0 being no damage, 1 damage < 20%, 2 damage 21%–40%, 3 damage 41%–60%, 4 damage 61%–80%, 5 damage > 80%, and 6 being dead plant. For RNA-seq, the samples (a whole plant above soil) were collected from the two genotypes at 0 (no aphid, control), 3, 6, 9 and 12 dpi. Samples were collected at the same time, and each treatment had three biological replicates that were pooled and frozen in liquid nitrogen and then transferred to a −80°C freezer where they were stored.

### 2.2 RNA isolation and transcriptome sequencing

For RNA isolation, seedling tissues were ground into a fine powder in liquid nitrogen and total RNA was extracted from each sample using TRIzol reagent (Invitrogen, Carlsbad, CA) as described by the manufacturer’s protocol. The RNA from each sample was treated with RNase-free DNase I (Invitrogen) to remove any contaminating DNA. Nanodrop™ 2000 spectrophotometer and agarose gel electrophoresis were used to check and confirm quantity and integrity of RNAs. RNA-seq libraries were constructed on the basis of the service from Novogene Corporation Inc. (Sacramento, CA, https://en.novogene.com/). Transcriptome sequencing was performed in an Illumina NovaSeq platform (NovaSeq 6000) to generate paired end (2x150bp) reads.

### 2.3 RNA-seq analysis

The raw RNA-seq reads were processed through fastp to remove the low-quality reads and reads containing adapter and poly-N sequences to obtain high quality reads. These cleaned, high-quality reads were mapped to the latest version of the *S. bicolor* genome v3.1.1 available from Phytozome (https://phytozome-next.jgi.doe.gov/info/Sbicolor_v3_1_1) using HISAT2 software (Kim et al., 2015). The mapped reads were assembled using StringTie, and the subread program *featureCounts* (Liao et al., 2014) was used to count the read numbers mapped to each gene. A principal component analysis (PCA) was carried out to explore the statistical correlations between the two genotypes and time points using the PCA package in R software. The differential expression analysis was performed using the EdgeR package (Robinson et al., 2010) by comparing the infested samples to control for each genotype and time points. Those genes that showed log_2_ ≥ 2 or ≤ −2 with a false discovery rate (FDR) adjusted p-value < 0.05 were considered as differentially expressed genes (DEGs). The other genes that showed low levels were removed, and only the DEGs were used for further analysis. The workflow for the RNA-seq analysis is depicted in **Supplementary Figure S2.**

### 2.4 Gene ontology and KEGG pathway analysis

The overlaps between different sets of DEGs were generated with bioinformatics and the evolutionary genomics online tool (https://bioinformatics.psb.ugent.be/webtools/Venn/). For functional annotation, Gene Ontology (GO) analysis was conducted using PlantRegMap tools (http://plantregmap.gao-lab.org/) with a threshold p-value of 0.05 to determine overrepresented GO categories in the up- and downregulated DEGs. Significantly enriched top 30 GO terms based on p-value were visualized in bar plots. Furthermore, for pathway analysis, the DEGs protein sequences were obtained by matching with those from the *S. bicolor* genome v3.1.1 (https://phytozome-next.jgi.doe.gov/). These protein sequences were used in the Kyoto Encyclopedia of Genes and Genomes (KEGG) database (BlastKOALA) to conduct pathway analysis. Initially, the pathways were selected on the basis of the GO enrichment in the resistant and susceptible genotypes. The information about the genes involved in the pathways was collected from the KEGG database and literature (Falcone Ferreyra et al., 2012) and the log2 fold change data were analyzed for these pathway-related genes. The BlastKOALA results gave information about the gene function and the pathway they belong to. Furthermore, literature was used to verify the functions of these genes and pathways during stress.

### 2.5 RNA extraction and quantitative real-time PCR analysis

Plant samples (whole seedling above soil) were collected from the two genotypes infested with sugarcane aphids and without infestation (control) at 0, 3, and 6 dpi. Each sample had three biological replicates for each time point and were frozen immediately in liquid nitrogen and stored at −80°C. The control samples were collected at each time point to eliminate the circadian rhythm effect on gene expression. TRIzol reagent (Invitrogen, Carlsbad, CA) was used to extract the total RNA from 100 mg of seedling tissue in each sample, and, then, it was treated with DNase (Turbo DNA-free kit, Thermo Fisher Scientific, Waltham, MA). A total of 2.5 μg of RNA was reverse-transcribed using the GoScript reverse transcriptase kit (Promega, Madison, WI), and the resulted cDNA was diluted four-fold before it was used in the real-time quantitative PCR (RT-qPCR).

For RT-qPCR, the DEGs identified as important in the pathway analysis were selected for expression verification. The selection criteria for the genes are as follows: DEGs that showed higher upregulation in resistant and susceptible genotypes across the time points or DEGs that showed downregulation in the susceptible genotype across the time points. In addition, these genes should have a role in stress mechanisms described in sorghum or related crops. The primers for these selected genes were designed using the IDT DNA program (https://www.idtdna.com/PrimerQuest/Home/Index), which are listed in **Supplementary Table S1**. A sorghum housekeeping gene, *α-Tubulin* (*Sobic.001G107200*), was used as the internal control as described previously (Huang et al., 2022; Shrestha and Huang, 2022). RT-qPCR was performed on a Bio-Rad iCycler thermal cycler (Bio-Rad Laboratories, Inc., Hercules, CA, USA) using the iTaq™ universal SYBR® green supermix (Bio-Rad Laboratories, Inc.). The RT-qPCR reaction was performed in a volume of 10 μl, containing 1 μl of cDNA, 0.4 μl (10 μM) each of the reverse and forward primers, 5 μl of SYBR green master mix, and 3.2 μl of ddH2O under the following conditions: one cycle at 95°C for 3 m, 40 cycles at 95°C for 10 s and 55°C for 30 s, followed by one cycle each of one min at 95°C and 55°C. The final melting curve was 81 cycles at 55°C for 30 s. The correlation of the selected DEGs was performed between RNA-seq data and RT-qPCR results, which was determined by Pearson’s correlation coefficient.

### 2.6 Phytohormone treatments

To further confirm the role of SA, JA, and abscisic acid (ABA) in plants in response to aphid infestation, a small population test was performed. Around 20–25 plants of two genotypes (BTx623 and Tx2783) were planted in a pot. At the two- to three-leaf stage (8–10 days), seedlings were sprayed with 600 μM SA (Le Thanh et al., 2017), 100 μM MeJA (Ma et al., 2020), and sterile distilled water (ddH_2_O, control) mix with 0.1% of Tween 20 to each pot separately, and each treatment had three replicates. After spraying, the pots were covered with a transparent cylindrical cage with nylon mesh on the top. Six hours after spraying, each pot was infested with 400 aphids. At 14 dpi, the plant mortality rate was recorded from each pot. In addition, aphid numbers on plants and plant damage scores were recorded from four random plants per pot at 14 dpi.

### 2.7 Statistical analysis

To assess differential responses from the plant-aphid interaction, aphid numbers on resistant and susceptible lines were recorded and compared, and the t-test was used to estimate the significant difference between the two genotypes. The t-test was also used to calculate any significant differences between aphid-infested and control samples (*P < 0.05 and **P < 0.01). For the phytohormone assay, the one-way ANOVA and Tukey test were used to determine the significant difference between treatments. For RT-qPCR, the relative expression level of each gene was calculated using the 2^−ΔΔCt^ method (Livak and Schmittgen, 2001), and the data used for this method are from SCA infested and control groups with three biological replicates. Each biological replicate value is the mean of two technical replicates.

## 3 Results

### 3.1 Differential responses between the resistant and susceptible genotypes

**Figure 1:**
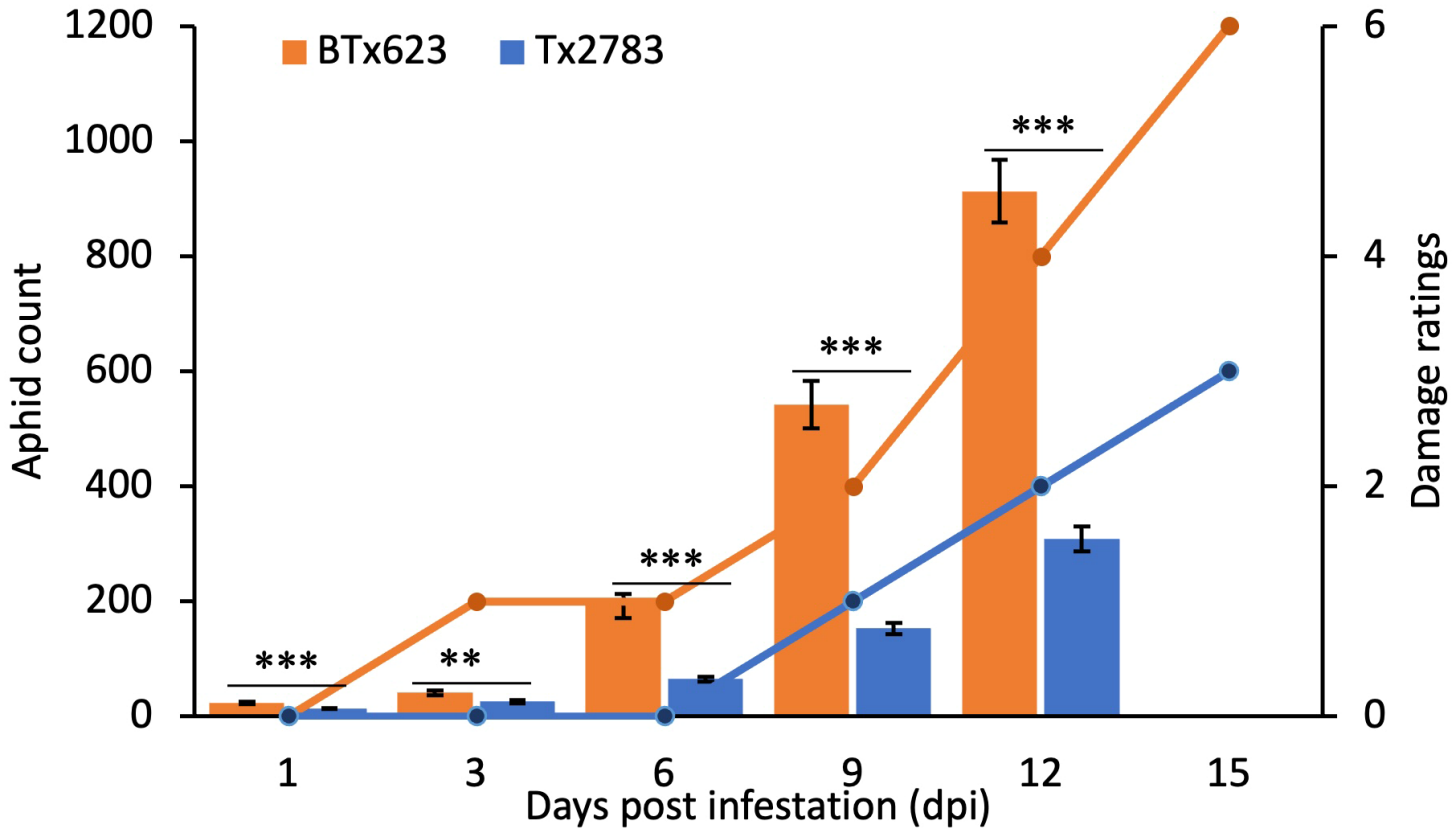
Aphid count and plant damage ratings data for two sorghum genotypes at a series of time points following sugarcane aphid infestation. The aphid count in each genotype is the mean of 10 plant samples ± standard error. The bar graph represents the average aphid count and line graph represents the damage ratings. The asterisk (*) at the *p-*value represents the significant difference between the genotype at the same time points (**P < 0.01, and ***P < 0.01: T-test).

Plants of resistant Tx2783 and susceptible BTx623 genotypes infested with sugarcane aphid showed differential responses to aphid infestation. Based on the phenotypic evaluation (**Figure 1**; **Supplementary Figure S1**), aphids caused minor damage to Tx2783 but severe plant damage to BTx623. The damage on Tx2783 scored 0 until 6 dpi (early time points), and the maximum damage score was 2 at 15 dpi. However, BTx623 plants were dying or completely dead by 15 dpi (**Figure 1**; **Supplementary Figure S1**). During the co-culture, Tx2783 showed an adverse effect on aphid development and fecundity in comparison with BTx623. The average number of aphids per plant suggested that the rate of aphid regeneration was significantly reduced on Tx2783 from early dpi (1 and 6 dpi) to late (9 to 12 dpi) in comparison with BTx623 (**Figure 1**). These results confirmed that Tx2783 was resistant to sugarcane aphids and that BTx623 was susceptible.

### 3.2 Analysis of RNA-seq data

**Figure 2:**
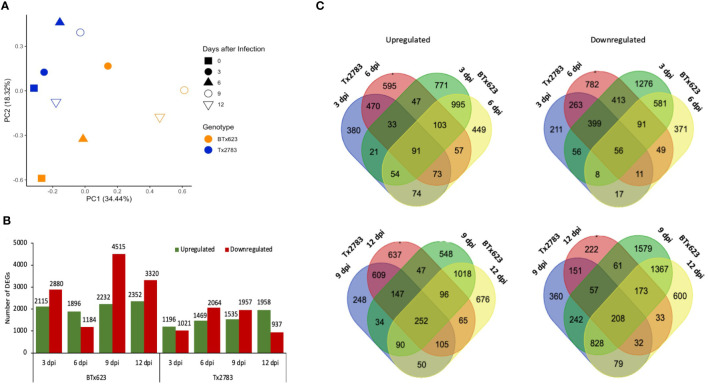
Summary of RNA-seq data of resistant Tx2783 and susceptible BTx623 sorghum genotypes infested with sugarcane aphid at different time points. **(A)** Principal component analysis of the two genotypes with various treatments. **(B)** Number of differentially expressed genes (DEGs); columns represent the number of genes upregulated and downregulated following exposure to the aphids compared to the controls. DEGs were defined as having a log2 fold change ≥ 2.0 or ≤ 2.0 with a false discovery rate (FDR) adjusted p-value < 0.05. **(C)** Venn diagrams of DEGs compared separately between 3 dpi vs. 6 dpi and 9 dpi vs. 12 dpi. The left is upregulated DEGs, and right is downregulated DEGs.

To explore the transcriptomic profile of sorghum in response to sugarcane aphid infestation, we performed RNA-seq analysis on a pair of sorghum genotypes, resistant Tx2783 and susceptible BTx623. The pair-end sequencing of RNA-seq libraries generated an average of 53.3 million good quality reads from individual samples (39.1–59.6 million reads). Among them, 47.8 million reads (36.9–47.4 million, 89.80%) on average were uniquely mapped to the sorghum reference genome v3.1.1 as shown in **Supplementary Table S2**. The PCA effectively separated resistant and susceptible genotypes from each other (**Figure 2A**). In PCA analysis, components 1 and 2 explained 52.76% of the variability. The first principal component accounting for 34.44% of the variance indicated the differential response between the resistant and susceptible genotypes. Similarly, the second component accounting for 18.32% of the variance, indicated the differential response between different time points. All the Tx2783 samples were seen in closed clusters (blue), separating from BTx623 samples (orange). The clustering of the samples according to the genotype indicates the metabolic and genetic diversity between the genotypes.

### 3.3 Dynamics of differential gene expression

The DEGs were identified for each time point and genotype by comparing aphid-infested samples to the control. Among four time points, 9 dpi of the susceptible genotype showed the highest number of DEGs (6,747) followed by 12 dpi (5,672) (**Supplementary Table S3**). Similarly, in the resistant line, Tx2783, 6 dpi (3,533) showed a higher number of DEGs followed by 9 dpi (3,492) (**Supplementary Table S3**). The DEGs were higher in the susceptible genotype, and, particularly, the numbers of the downregulated DEGs were much higher in the susceptible genotype in comparison with that in the resistant genotype (**Figure 2B**). In contrast, the resistant genotype showed a gradual increase of the upregulated DEGs sequentially and decrease in the downregulated DEGs. Furthermore, Venn diagrams were constructed to show the overlaps between different sets of DEGs in the early time points (3 and 6 dpi) and late time points (9 and 12 dpi) (**Figure 2C**). In early time points, both the upregulated and downregulated Venn diagrams showed a greater number of unique DEGs in each genotype in comparison with that of the shared DEGs (**Supplementary Table S4**). Similar results were observed in late time points, except in downregulated genes in the resistant genotype. The higher number of unique DEGs in aphid-infested plants suggests that the resistant and susceptible genotypes underwent different genetic and metabolic changes to confront aphid attack.

### 3.4 Gene ontology enrichment of DEGs

**Figure 3:**
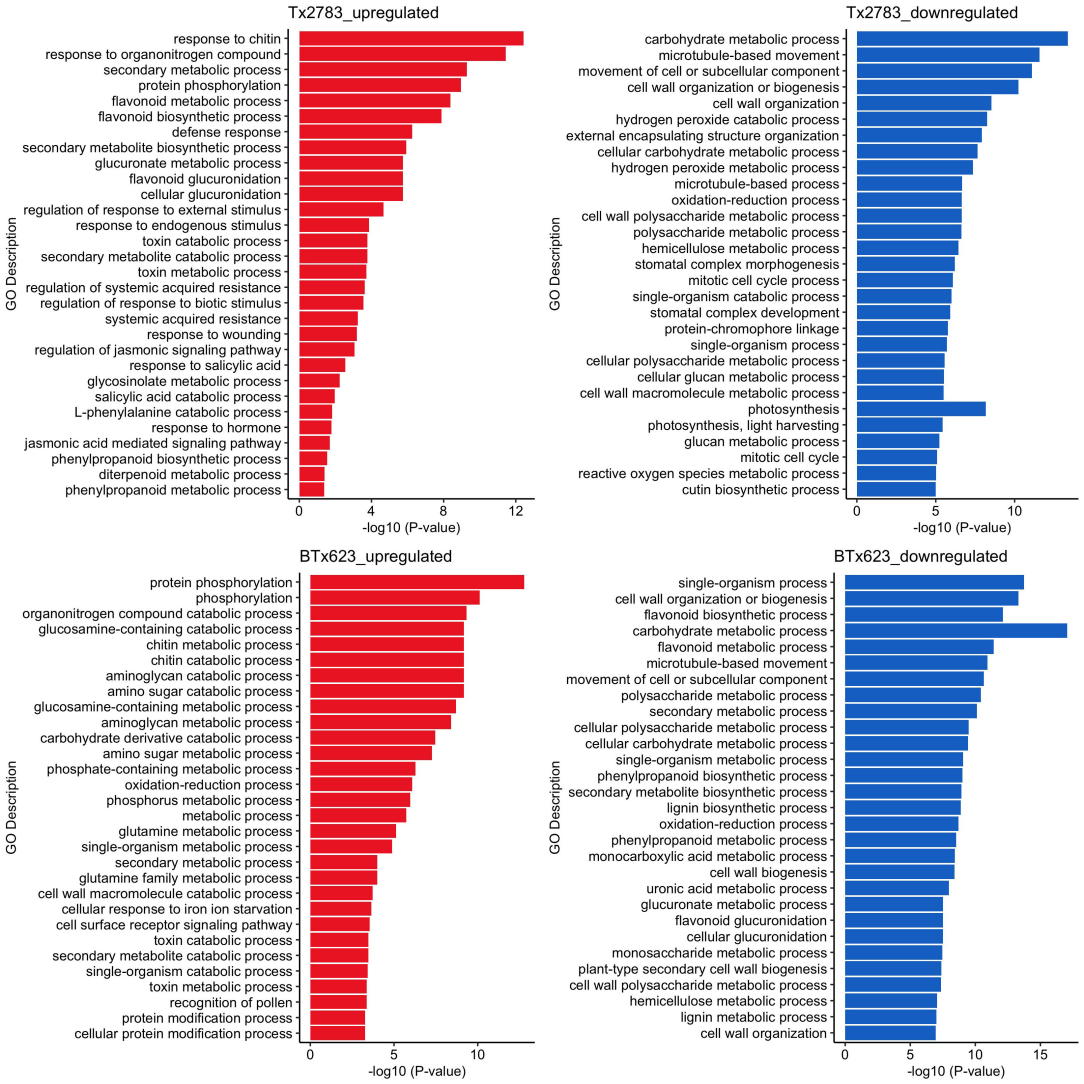
Gene ontology (GO) terms of DEGs of resistant Tx2783 and susceptible BTx623 sorghum genotypes exposed to sugarcane aphids. All the DEGs of four time points were combined, and unique genes of each genotype were used for GO analysis. The top 30 GO terms based on *P*-value are represented in bar plots, each bar represents the −log_10_ (*P* < 0.05) of an individual GO term and a longer bar reflects the most significant GO terms.

Gene enrichment analysis based on gene annotation was conducted to comprehend the biological and molecular functions of DEGs in each genotype. All expression data for four time points were combined, and unique genes were identified for each genotype as shown in **SupplementaryTables S5** and **S7** (Tx2783 upregulation, Tx2783 downregulation, BTx623 upregulation, and BTx623 downregulation, respectively) and plotted in bar plots (**Figure 3**). In Tx2783 upregulation, the GO terms related to defense response, JA and SA regulation, response to hormone, and wounding were found. Similarly, the GO terms like secondary metabolic process and flavonoid metabolic and biosynthesis process were also enriched here. In contrast, the BTx623 downregulation had GO terms related to secondary metabolic process, flavonoids, and lignin metabolic process. The Tx2783 downregulation was enriched with GO terms related to cell wall organization and photosynthesis. Interestingly, the flavonoid biosynthesis process was noted as upregulation in Tx2783, but downregulation in BTx623.

### 3.5 KEGG analysis and specific aphid-related pathways and genes

KEGG annotation and pathway analysis was conducted for all four time points: the number of annotated DEGs and unique pathways gradually increased with the time points in Tx2783 upregulated DEGs (**Supplementary Table S6**). As described in the methods section, first the GO-enriched pathways were selected from GO analysis. Those pathways that had multiple pathway-related DEGs upregulated in the resistant genotype and up- or downregulated in the susceptible genotype were considered important in plant defense against sugarcane aphids. In our study, plant hormone pathways for biotic stresses like jasmonic acid (JA) and phenylpropanoid were upregulated in the resistant genotype (**Supplementary Table S8**). Similarly, the NLR proteins, MAPK signaling, and transcription factors (TFs) were also differentially expressed in the two genotypes. The important metabolites for plant defense, flavonoids, terpenoids, and glutathione were also upregulated in the resistant genotype. Based on the GO and KEGG enrichment pathway analysis, these identified pathways and defense-related genes have a potential role in sorghum defense against sugarcane aphids. These aphid responsive genes in plants were grouped according to their functions and discussed in detail.

### 3.6 NLR genes and signaling pathway genes

**Figure 4:**
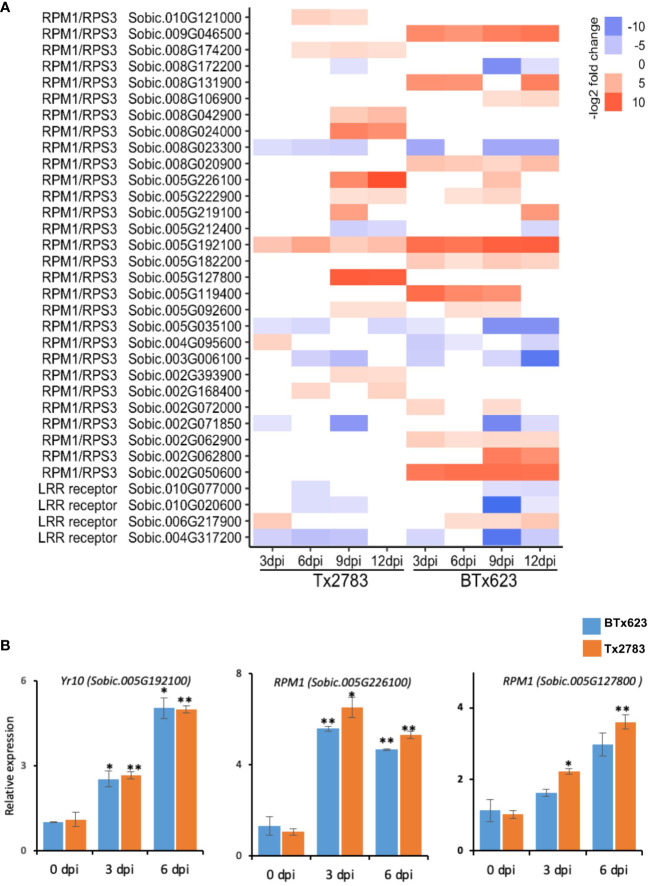
**(A)** The heatmap represents transcript abundance of the resistant Tx2783 and susceptible BTx623 genotypes in response to sugarcane aphid infestation relative to the controls for NLR gene. **(B)** RT-qPCR was used to confirm the relative expression of selected genes, and it was calculated using the 2^−ΔΔCt^ method. Each error bar represents the ± standard error (n = 3), and asterisks indicate significant differences between the control and aphid infested samples, *P < 0.05 and **P < 0.01. *RPM1/RPS3*, *Resistance to P. syringae* pv. *maculicola 1/Resistance to Pseudomonas syringae 3*; *LRR receptor*, *leucine-rich repeat receptor–like serine/threonine protein kinase*; *Yr10*, *Stripe rust resistance protein Yr10*.

Upon wounding by aphids, the plant recognizes the effectors of aphids through pattern recognition receptors like NLR proteins. In total, 33 NLR genes were differentially expressed in comparison with control (**Figure 4A**), and these genes belong to two families; *RPM1/RPS3* (*Resistance to P. Syringae* pv*. maculicola 1*, *Resistance to Pseudomonas syringae 3*) and *LRR receptor* (*Leucine-rich repeat receptor*) (**Figure 4B**). Most genes of the *RPM1/RPS3* family were upregulated in the resistant line in late time points (9 and 12 dpi), whereas the genes of the LRR receptor were upregulated at 3 dpi only and highly downregulated in the susceptible genotype across all time points. The NLR gene *Sobic.005G192100* (identical to the *Yr10*) was upregulated at all time points across both genotypes (Liu et al., 2014). The other NLR genes upregulated across multiple time points and in both genotypes were *Sobic.005G226100*, *Sobic.005G127800*, *Sobic.005G222900*, and *Sobic.005G092600*. The RT-qPCR results (**Figure 4B**) showed significantly higher expression of these two NLR genes (*Sobic.005G192100* and *Sobic.005G226100*) in both genotypes across 3 and 6 dpi.

**Figure 5:**
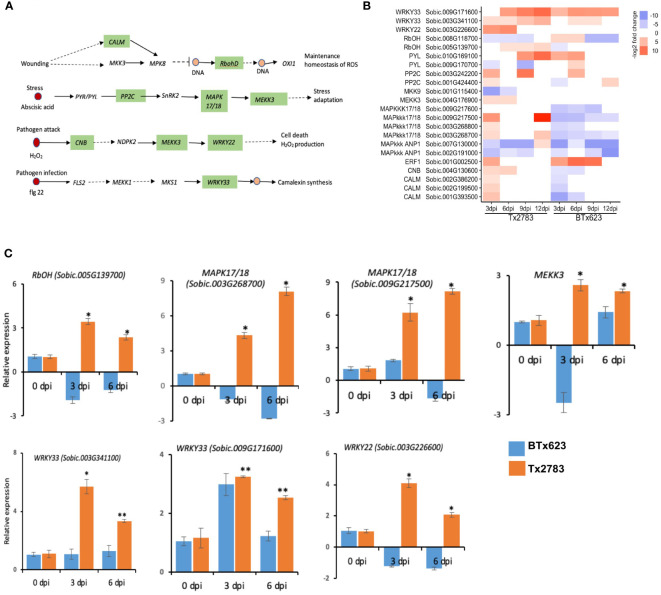
Effect of sugarcane aphid feeding on expression of the MAPK signaling pathway. **(A)** Schematic diagram of the MAPK signaling pathway (dotted arrow represents multiple enzymatic steps, and the green highlighted enzymes were identified with differential expression during aphid infestation). **(B)** The heatmap represents transcript abundance of the resistant Tx2783 and susceptible BTx623 genotypes in response to sugarcane aphid infestation relative to the controls. **(C)** RT-qPCR was used to confirm the relative expression of the selected genes and it was calculated using the 2^−ΔΔCt^ method. Error bars represent the ± standard error (n = 3), and asterisks indicate significant differences between the control and aphid infested samples, *P < 0.05 and **P < 0.01. *CALM*, *Calmodulin: RbohD*, *Respiratory burst oxidase homolog protein; PP2C*, *Protein phosphatase 2C*; *MAPK17/18*, *Mitogen-activated protein kinase kinase kinase 17/18*; *MEKK3*, *Mitogen activated protein kinase 3*; *CNB*, *Serine/threonine-protein phosphatase*; *WRKY*, *WRKY transcription factor*.

Induction of MAPKs was the earliest signaling event following herbivore attacks, suggesting their important role in signal amplification. A detail of the MAPK pathways is illustrated in **Figure 5A**, where each pathway leads to the production of ROS and maintenance, stress adaptation, cell death, or camalexin synthesis. The important genes in these pathways are *mitogen-activated protein kinase 17/18* (*MAPK 17/18*), *WRKY transcription factor* (*WRKY*), *Calmodulin* (*CALM*), and *Respiratory burst oxidase homolog protein* (*RbOHD*). Three genes of the *MAPK* 17/18 family (*Sobic.003G268700*, *Sobic.003G268800*, and *Sobic.009G217500*) were upregulated in the resistant genotype at 3 and 12 dpi but downregulated in the susceptible genotype (**Figure 5B**), which were also supported by the RT-qPCR data of 3 and 6 dpi (**Figure 5C**). The other *MAPKs*, *MEKK3* and *Sobic.004G176900*, were also upregulated in the resistant genotype at early dpi. The RT-qPCR data also supported the upregulation of *MEKK3* in the resistant genotype at 3 and 6 dpi (**Figure 5C**). Similarly, *WRKY22* (*Sobic.003G226600*) and *WRKY33* (*Sobic.003G341100* and *Sobic.009G171600*) genes were upregulated in both genotypes across all time points. Similar significant upregulation was detectable in the RT-qPCR data in the resistant genotype.

### 3.7 Plant hormones and signal transduction

**Figure 6:**
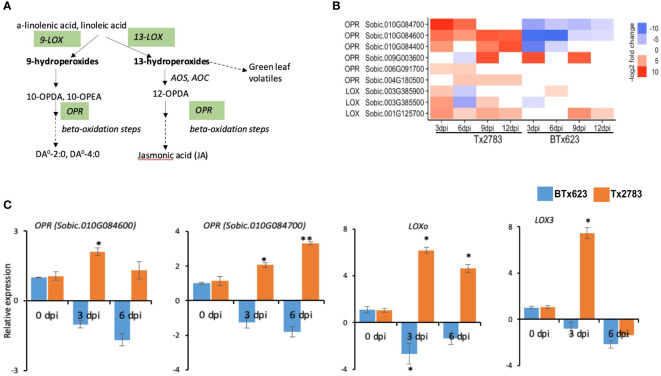
Effect of sugarcane aphid feeding on expression of the genes in the linolenic acid linolenic acid pathway. **(A)** Schematic diagram of linolenic acid pathways is modified from Chrsitensen et al. (2015); JA linolenic acid pathways (dashed arrow represents multiple enzymatic steps, and the green highlighted enzymes were identified with differential expression during aphid infestation). **(B)** The heatmap represents transcript abundance of the resistant Tx2783 and susceptible BTx623 genotypes in response to sugarcane aphid infestation relative to the controls. **(C)** RT-qPCR was used to confirm the relative expression of the selected genes, and it was calculated using the 2^−ΔΔCt^ method. Error bars represent the ± standard error (n = 3), and asterisks indicate significant differences between the control and aphid infested samples, *P < 0.05 and **P < 0.01. *LOX*, *lipoxygenase*; *AOS*, *allene oxide synthase*; *AOC*, *allene oxide cyclase*; *OPR*, *12-oxophytodienoic acid reductase*; 10-OPEA, 10-oxo-11-phytoenoic acid; 10-OPDA, 10-oxo-11,15-phytodienoic acid; 12-OPDA, 12-oxo-10,15-phytodienoic acid; DA, Death acids; DA^0–^4:0, 4-[(1,5)-2-oxo-5-pentylcyclopent-3-ene-1-yl] butanoic acid; DA^0–^2:0, (2-[(1, 5)-2-oxo-5-pentylcyclopent-3-ene-1-yl] acetic acid.

Plant hormones are regulators of almost all aspects of plant development and plant responses to the environment (Foo et al., 2019). JA is synthesized from the linolenic acid pathway (**Figure 6A**); in addition to JA, this pathway also synthesizes defense compounds like oxylipins and death acids. JA is derived from linolenic acid via an octadecanoid pathway, and the precursor linolenic acid also forms oxylipins (death acids and green leaf volatiles) (**Figure 6A**). Our study identified 10 DEGs in this pathway (**Figure 6B**), and they belong to two gene families, *lipoxygenase* (*LOX*) and *12-oxophytodienoic acid reductase* (*OPR*). Three *LOX* genes belong to 9-S-LOXs, and they are responsible for the production of death acids (Christensen et al., 2015; Shrestha et al., 2021). The RT-qPCR results of *SbLOXo* (*Sobic.001G125700*) and *SbLOX3* (*Sobic.003G385500*) were consistent with both RNA-seq analysis (**Figure 6C**) at the early time points. An *OPR* gene of JA pathway converts 12-oxoophytodieonic acid (OPDA) into JA through multiple steps of beta-oxidation (**Figure 6A**). In this study, seven differentially expressed *OPR* genes were identified, of which two *OPR* genes, *Sobic.010G084600* and *Sobic.010G084700*, were upregulated in the resistant genotype and downregulated in the susceptible genotype across the time points. The RT-qPCR result of these two *OPR* genes showed similar expression patterns (**Figure 6C**).

**Figure 7:**
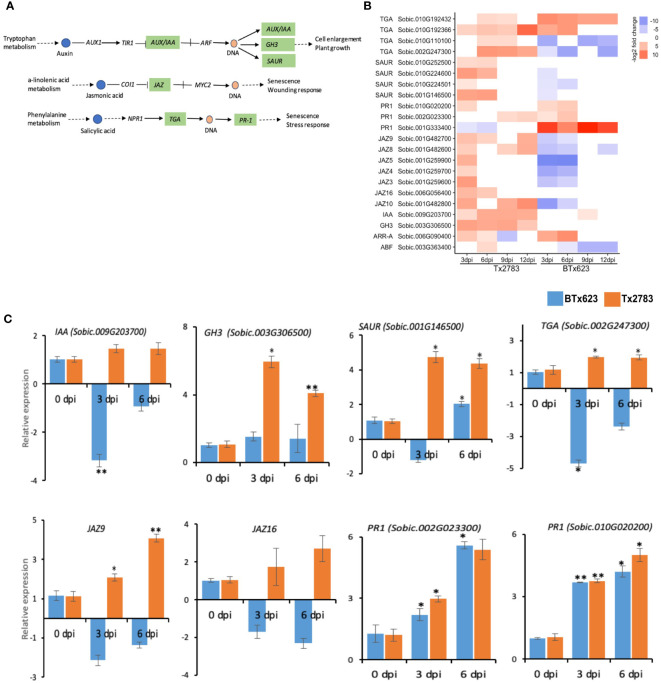
Effect of sugarcane aphid feeding on the plant hormone signal transduction pathway. **(A)** Schematic diagram of the plant hormone signal transduction pathway (dotted arrow represents multiple enzymatic steps, the green highlighted enzymes were identified with differential expression in response to aphid infestation. **(B)** The heatmap represents transcript abundance in resistant Tx2783 and susceptible BTx623 genotypes after aphid infestation relative to the controls. **(C)** RT-qPCR confirmation of the relative expression of the selected pathway genes, which was calculated using the 2^−ΔΔCt^ method. Error bars represent the ± standard error (n = 3) and asterisks indicate significant differences between the control and the aphid infested samples, *P < 0.05 and **P < 0.01. The bars without asterisk are nonsignificant (*P* > 0.05). *AUX*, *auxin influx carrier*; *AUX/IAA*, *auxin/indole3acetic acid*; *GH3*, *auxin responsive Gretchen hagen3*; *SAUR*, *small auxin upregulated RNA*; *JAZ*, *jasmonate ZIM-domain*; *TGA*, *transcription factor TGA*; *PR1*, *pathogenesis related1*.

In the hormone signal transduction pathway, 22 genes were differentially expressed in the two genotypes in response to aphid infestation (**Figure 7B**). These genes belong to auxin, JA, and SA signal transduction pathways (**Figure 7A**). Auxin is involved in the regulation of cell growth and plant development, and it is also involved in regulating the host defense signaling and resistance mechanisms (Kazan and Manners, 2009). The RNA-seq revealed upregulation of *AUX/IAA* (*auxin/indole-3-acetic acid*; *Sobic.009G203700*), *GH3* (*auxin-responsive Gretchen hagen3*; *Sobic.003G306500*), and *SAUR* (*small auxin upregulated RNA*; *Sobic.010G224600*) in the resistant genotype, and their upregulation was confirmed by the RT-qPCR data as well (**Figure 7C**). In the JA signal transduction, seven genes from the *jasmonate ZIM-domain* (*JAZ*) gene family were differentially expressed in the two genotypes. Almost all these seven genes were upregulated in the resistant genotype at 3 dpi but downregulated in the susceptible genotype. All these seven sorghum *JAZ* genes of them were reported in the previous study (Shrestha and Huang, 2022). RT-qPCR data of *SbJAZ9* and *SbJAZ16* showed an upregulation in the resistant genotype at early time points (**Figure 7C**). As for the SA signal transduction pathway, four genes encoding the transcription factor TGACG-binding site (TGA) were upregulated in the resistant genotype (**Figure 7B**). Among the four *TGA* genes, *Sobic.002G247300* showed differential expression over all time points based on the data from RNA-seq and RT-qPCR (**Figures 7B, C**). During SA production, the TGA acts synergistically with nonexpresser *PR* gene1 (*NPR1*) and regulates the *pathogenesis-related* (*PR*) genes that induce systemic acquired resistance (Kesarwani et al., 2007). Among these three *PR-1* genes, two of them (*Sobic.002G023300* and *Sobic.010G0202000*) were upregulated in both genotypes across the time points (**Figure 7B**). The RT-qPCR results of these two *PR-1* genes also showed similar patterns in both genotypes across the time points (**Figure 7C**).

### 3.8 Plant secondary metabolites

**Figure 8:**
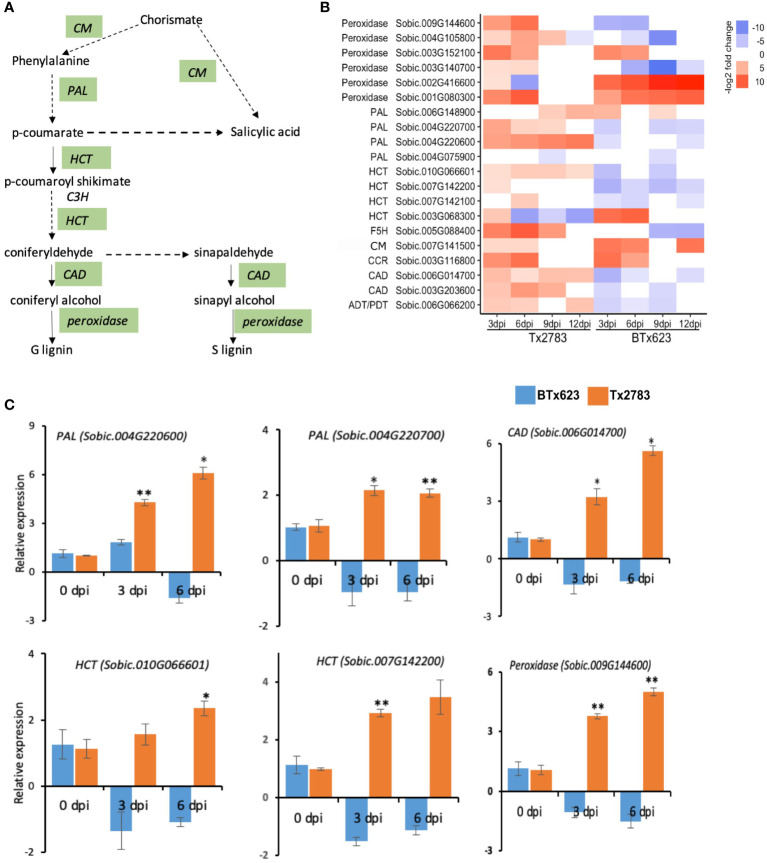
Effect of sugarcane aphid feeding on phenylpropanoid pathways gene expression. **(A)** Schematic diagram of phenylpropanoid pathways (dashed arrow represents multiple enzymatic steps, and the green highlighted enzymes are expressed during aphid infestation). **(B)** The heatmap represents transcript abundance for resistant Tx2783 and susceptible BTx623 genotypes after aphid infestation relative to control samples. **(C)** RT-qPCR was used to confirm the relative expression of selected genes, and it was estimated using the 2^−ΔΔCt^ method. Error bars in each bar represent the ± standard error (n = 3), and asterisks indicate significant differences between the control and aphid infested samples, *P < 0.05 and **P < 0.01. *CM*, *chorismite mutase*; *PAL*, *phenylalanine ammonia-lyase*; *C4H*, *cinnamate 4-hydroxylase*; *HCT*, *shikimate O-hydroxy cinnamoyl transferase*; *C3H p-coumarate 3-hydroxylase*; *CAD*, *cinnamyl-alcohol dehydrogenase*; ADT/PDT, arogenate/prephenate dehydratase.

During attack by pests, plant secondary metabolites (PSMs) accumulate in elevated levels, which will serve as defense compounds against herbivores and pathogens (Wink, 2018). The important PSM pathway and related genes observed in our study are phenylpropanoid, flavonoid, and terpenoid biosynthesis pathways. In the phenylpropanoid pathway (**Figure 8A**), a total of 20 genes were differentially expressed in the two genotypes; most of them were upregulated in the resistant genotype (**Figure 8B**). Of which, four *phenylalanine ammonia-lyase* (*PAL*) genes were differentially expressed. Among them, two (*Sobic.004G220700* and *Sobic.004G220600*) were increased more than four-fold in the resistant genotype (**Figure 8B**). The RT-qPCR data of both *PAL* genes (**Figure 8C**) further confirmed the upregulation of the genes in the resistant genotype and downregulation in the susceptible genotype. *PAL* catalyzes the conversion of phenylalanine to cinnamic acid, the key step in this pathway that ultimately forms lignin, flavonoid, and SA (**Figure 8A**) (Lv et al., 2017). SA biosynthesis also starts from the chorismate using the isochorismate pathway (ICS) (**Figure 8A**) (Shine et al., 2016). *Chorismate mutase* (CM) that was upregulated in both genotypes helps in SA biosynthesis through both PAL and ICS pathways. The other genes, *shikimate O-hydroxy cinnamoyl transferase* (*HCT*) and *cinnamyl-alcohol dehydrogenase* (*CAD*), are important for lignin formation. In total, three *HCT* genes (*Sobic.007G142100*, *Sobic.010G066601*, and *Sobic.007G142200*) and two *CAD* genes (*Sobic.006G014700* and *Sobic.003G203600*) were upregulated in the resistant genotype and downregulated in the susceptible one (**Figure 8B**), which were further validated by RT-qPCR data (**Figure 8C**).

**Figure 9:**
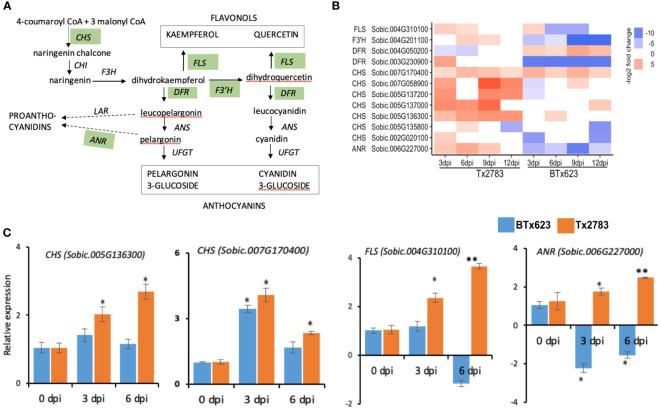
Effect of sugarcane aphid feeding on expression of the flavonoid pathway genes. **(A)** Schematic diagram of the flavonoid pathway is modified from Falcone Ferreyra et al. (2012) (dotted arrow represents multiple enzymatic steps, and the green highlighted enzymes were expressed in plants during aphid infestation). **(B)** The heatmap represents transcript abundance for resistant Tx2783 and susceptible BTx623 genotypes after aphid infestation relative to control samples. **(C)** RT-qPCR was used to confirm the relative expression of the selected genes, and it was estimated using the 2^−ΔΔCt^ method. Error bars in each bar represent the ± standard error (n = 3) and asterisks indicate significant differences between the control and aphid infested samples, *P < 0.05 and **P < 0.01. *CHS*, *chalcone synthase*; *CHI*, *chalcone isomerase*; *F3′H*, *flavonoid 3-hydroxylase*; *DFR*, *dihydroflavonol 4-reductase*; *FNR*, *flavanone 4-reductase*; *ANS*, *anthocyanidin synthase*; *UFGT*, *UDPglucose flavonoid 3O glucosyltransferase*; *FLS*, *flavonol synthase*; *LAR*, *leucoanthocyanidin reductase*; *ANR*, *anthocyanidin reductase*.

**Figure 10:**
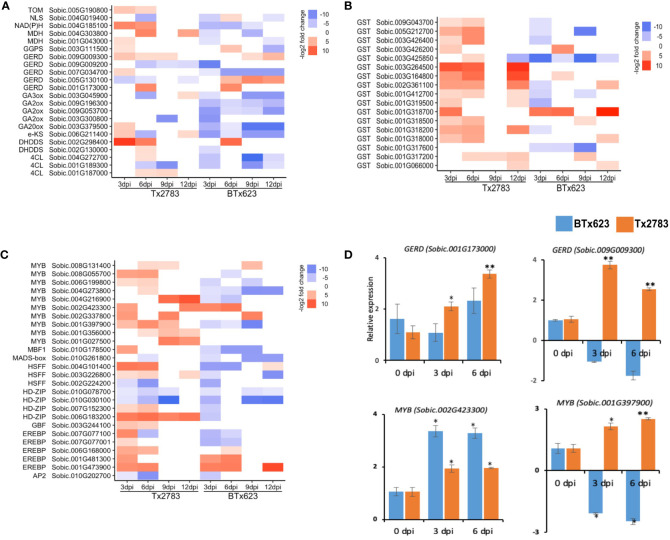
The heatmap represents transcript abundance in both resistant Tx2783 and susceptible BTx623 genotypes after aphid infestation relative to the control samples. The transcripts are related to terpenoids **(A)**, glutathione **(B)**, and transcription factors **(C)**. **(D)** RT-qPCR was used to confirm the relative expression of selected genes, and it was estimated using the 2^−ΔΔCt^ method. Error bars in each bar represent the ± standard error (n = 3), and asterisks indicate significant differences between the control and aphid infested samples, *P < 0.05 and **P < 0.01. *GERD*, *(*−*)germacrene D synthase*; *GGPS*, *geranylgeranyl diphosphate synthase*; *GA20ox*, *gibberellin44 dioxygenase*; *eKS*, *ent-kaurene synthase*; *NLS*, *nerolidol synthase*; *DHDDS*, *ditrans*, *polycis-polyprenyl diphosphate synthase*; *MDH*, (*+*)*neomenthol dehydrogenase*; *NAD*(*P*)*H*, *dehydrogenase* (*quinone*); *TOM*, *tocopherol O-methyltransferase*; *GST*, *glutathione S-transferase*; EREBP, ethyelene-responsive element binding proteins; HDZIP, homeobox leucine zipper protein; HSFF, heat shock transcription factor; AP2, AP2like factor; MYB, transcription factor MYB; MADS, MADS-box transcription factor.

PSMs, such as flavonoids, are also synthesized through the phenylpropanoid pathway, transforming phenylalanine into 4-coumaroyl-CoA (Falcone Ferreyra et al., 2012). The first enzyme for the flavonoid pathway is chalcone synthase (CHS), which produces chalcone scaffolds from which all flavonoids derive (**Figure 9A**). In our study, seven *CHS* genes were upregulated in the resistant genotype. Among them, five *CHS* genes (*Sobic.005G136300*, *Sobic.005G137000*, *Sobic.007G170400*, *Sobic.007G058900*, and *Sobic.005G137200*) were upregulated more than four-fold across the time points in the resistant genotype compared to susceptible (**Figure 9B**). The expression analysis from RT-qPCR for two of the genes (*Sobic.005G136300* and *Sobic.007G170400*) further supported this upregulation in the resistant genotype (**Figure 9C**). In addition, PSM terpenes and terpenoids are the largest group of secondary metabolites and provide plant defense through feeding deterrence, direct toxicity, or oviposition deterrence (Divekar et al., 2022). In total, 15 DEGs were identified as terpene biosynthesis-related genes in the sorghum lines infested with aphids (**Figure 10A**). We found five DEGs of (−)-germacrene D synthase (GERD), and, among those, three (*Sobic.007G034700*, *Sobic.009G009300*, and *Sobic.001G173000*) were upregulated more than two-fold in the resistant genotype compared to susceptible (**Figure 10A**). The RT-qPCR data (**Figure 10D**) from two of the *GERD* genes (*Sobic.001G173000* and *Sobic.009G009300*) showed a higher expression in the resistant genotype, which supports the RNA-seq data.

Glutathione is a sulfur-containing PSM that has an antioxidant function through involvement in cell redox homeostasis (Dubreuil-Maurizi and Poinssot, 2012). The glutathione-s-transferase (GST) is regarded as the marker gene for the oxidative stress along with respiratory burst oxidase Homolog D (*RbOHD*) and peroxidase (Dubreuil-Maurizi and Poinssot, 2012). Our study shows that 15 genes from the *GST* family were upregulated in the resistant genotype. Among them, 11 were upregulated more than two-fold at 3, 6, and 12 dpi (**Figure 10B**). Another antioxidant gene, *peroxidase*, in the phenylpropanoid pathway codes for ROS-detoxifying enzymes and regulates the redox and Ca^2+^ homeostasis (Kawano, 2003; Gulsen et al., 2010). Six genes from the peroxidase family were highly upregulated at early time points of the resistant genotype following aphid infestation (**Figure 8B**). Among them, three *peroxidase* genes (*Sobic.009G144600*, *Sobic.003G140700*, and *Sobic.004G105800*) were upregulated in the resistant plants but downregulated in the susceptible plants, whereas two other *peroxidase* genes (*Sobic.001G080300* and *Sobic.003G152100*) were upregulated in both genotypes. The RT-qPCR results for one of the *peroxidase* genes (*Sobic.009G144600*) showed similar expression as the RNA-seq data (**Figure 8C**).

### 3.9 Transcription factors

Transcription factors (TFs) are key components that control gene expression in all living organisms. In our study, during aphid infestation, 24 TFs were differentially expressed in both sorghum genotypes over all time points (**Figure 10C**). The major TFs included ET-responsive element binding proteins (EREBP), homeobox-leucine zipper protein (HD-Zip), heat shock transcription factor (HSFF), and transcription factor MYB (MYB). Among four HD-Zip genes differentially expressed, one of them (*Sobic.006G183200*) was expressed nine-fold in the resistant genotype across all the time points (**Figure 10C**). Similarly, 10 MYB DEGs in sorghum during aphid infestation were noted. Among them, four MYB genes (*Sobic.006G199800*, *Sobic.001G397900*, *Sobic.008G055700*, and *Sobic.004G216900*) were highly upregulated in the resistant genotype but downregulated in the susceptible genotype, and two of them (*Sobic.002G423300* and *Sobic.002G337800*) were upregulated in both genotypes (**Figure 10C**). The expression analysis from RT-qPCR of the two genes (*Sobic.002G423300* and *Sobic.001G397900*) further supported their expression pattern that was revealed by RNA-seq in both resistant and susceptible genotypes (**Figure 10D**). In the other group of TFs, EREBP also plays a role in the hormone signal transduction pathway including ET, ABA, cytokinin, and JA (Shen et al., 2003; Rashotte and Goertzen, 2010; Hu et al., 2013). Five EREBP TF genes were also identified in the differentially expressed profiles of sorghum genotypes during aphid infestation. Among them, two EREBP (*Sobic.001G473900* and *Sobic.006G168000*) were upregulated more than six-fold across the time points in the two genotypes (**Figure 10C**). Furthermore, the Pearson correlation analysis of 36 DEGs were used for correlation analysis, and a high correlation was observed between RNA-seq and RT-qPCR data (R^2^ = 0.6017, r = 0.7757) (**Supplementary Figure S3**). The high correlation between the two analysis methods indicates that the measured changes in gene expression detected by RNA-seq reflect the actual transcriptomic difference between the two sorghum genotypes as previously reported (Kiani and Szczepaniec, 2018).

### 3.10 Effect of phytohormonal treatment on host defense in susceptible genotypes

**Figure 11:**
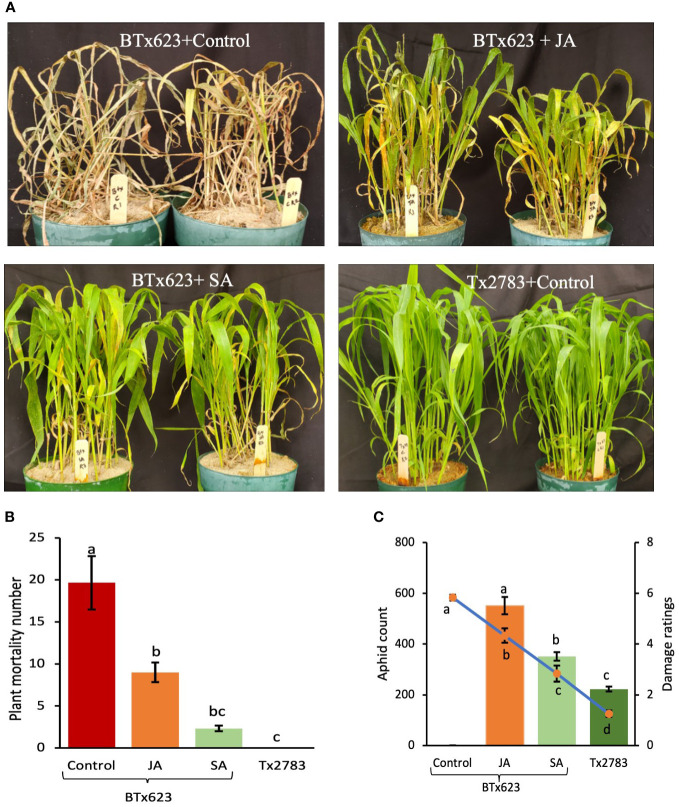
Phytohormone bioassay of susceptible BTx623 and resistant Tx2783 genotypes after 14 days of sugarcane aphid infestation. The BTx623 were treated with SA and JA, and the control samples of BTx623 and Tx2783 were sprayed with distilled water. All samples were treated with 400 aphids and were evaluated at 14 dpi **(A)** plant mortality and **(B)** aphid count and **(C)** damage were rated. Statistical analysis of plant mortality, aphid count, and damage ratings were done using one way ANOVA and Tukey tests. Error bars in plant mortality represent ± standard error (n=3) and in aphid count and damage ratings represent ± standard error (n = 12). The letter above the column indicates significant difference relative to each other (P < 0.05).

To further corroborate the positive role of JA and SA pathways in host plant defense, exogenous phytohormones (JA and SA) were applied to the experimental sorghum plants prior to aphid infestation. The phenotype of the plants treated with phytohormones and aphids corresponded to the plant mortality graph, aphid count, and damage score (**Figure 11**). The plant mortality at 14 dpi on different treatments were highly significant (p-value < 0.001). The control (BTx623, susceptible to aphids), sprayed with ddH_2_O, showed the highest mortality rate, whereas Tx2783 (the resistant genotype) control showed 0 dead plants with aphid infestation (**Figure 11A**). The BTx623 treated with JA showed nine dead plants, which was significantly less than the BTx623 control, whereas BTx623 treated with SA showed only three dead plants and was significantly at par with the Tx2783 treatment. Similarly, aphid count and damage score also showed high significant difference (p-value < 0.0001) (**Figure 11C**). The control BTx623 was almost all dead at 14 dpi with damage ratings of 6 (aphid count data were not recorded for this treatment). BTx623 treated with JA showed aphid count of 550 and a damage score of 4.3, whereas treated with SA showed significantly lower aphid count of 351 and a damage score of 2.8. Among all, Tx2783 control showed the lowest aphid count with 223 and a damage score of 1.25 and was significantly different from all other BTx623 treatments. Both the 100 μM JA and 600 μM SA treatments had a significant resistant effect on host plant response with SA having a more prominent effect on plant mortality, aphid count, and damage score.

## 4 Discussion

For a better understanding of the molecular interaction between sorghum plants and sugarcane aphid, aphid-resistant Tx2783 and aphid-susceptible BTx623 lines were compared in parallel to analyze molecular responses to the aphid. In this study, both sorghum genotypes were infested with the aphids and allowed to culture for a certain time, after which the Tx2783 plants showed a significantly lower aphid population and lower damage ratings in comparison with BTx623 supporting the antibiosis mechanisms of resistance (**Figure 1**). Previous studies have revealed that Tx2783 showed antibiosis and antixenosis mechanism of resistance against sugarcane aphids (Tetreault et al., 2019). The genotypes that possess more than one category of resistance are considered better because antibiosis suppresses the population of aphids and antixenosis deters or repels the pest (Paudyal et al., 2019). The molecular basis of sorghum resistance to aphids has been studied previously through a few RNA-seq studies. The first study on sorghum and sugarcane aphid interaction (Kiani and Szczepaniec, 2018) was focused on the late response of sorghum against sugarcane aphids with different resistant genotypes (DKS 44–20 Deklab, IL). The early time point study by Serba et al. (2021) used a different resistant line (TAM48) and was focused on sorghum TFs only. Similarly, the study by Puri et al. (2023) has also used a different resistant line (SC265). The research by Tetreault et al. (2019) used the similar resistant genotype (Tx2783), but the analysis lacks the comprehensive study of the pathway genes and TFs in detail. Therefore, our study is more focused toward the comprehensive analysis of pathway-related genes at both early and late time points and to further strengthen the role of these genes in aphid defense.

Our transcriptomic analysis of resistant and susceptible sorghum genotypes during aphid infestation revealed a suite of genes that were induced in both early (3 and 6 dpi) and late time points (9 and 12 dpi). The DEGs identified during HPR were involved in a series of molecular events that include signal perception (NLR genes), signal transduction (MAPK signaling and phytohormones), and defense response (TFs, flavonoids, and terpenoids). The host plants probably first recognized the elicitors and effectors of the aphids to deploy a constitutive defense as well as an induced defense, resulting in reprogramming of gene expression and biochemical pathways (Thompson and Goggin, 2006b; Meihls et al., 2012). Following elicitor recognition, the plant recognized the effectors of aphids through NLR proteins, which, in turn, activated ETI. A total of 33 NLR DEGs genes were identified (**Figure 4**). Among them, *Sobic.005G192100* belonging to the *RPM1/RPS3* gene family was upregulated more than four-fold in both genotypes across all the time points. This gene is the putative ortholog to *stripe rust resistance protein Yr10* in wheat and is highly conserved among monocots (Liu et al., 2014). In wheat, transformation of the *Yr10* gene in the susceptible line showed resistance against stripe rust. The *Yr10* gene encodes a coiled-coil (CC)-NLR protein that recognizes avirulent proteins and elicits hypersensitive reponse (Liu et al., 2014). Several NLR genes like *BROWN PLANTHOPPER RESISTANCE* (*BPH*) of rice (Du et al., 2009), *Mi-1.2* of tomato (Jesse et al., 1998) and *Vat* of melon (Dogimont et al., 2014) encode CC-NLR proteins to impart resistance against the pest. Future studies should focus on verifying the role of these two sorghum genes in aphid resistance.

After perception of effectors by NLR genes, signaling events of were the first to occur (Hettenhausen et al., 2015). During aphid infestation, three *MAPK 17/18* and one *MEKK3* genes involved in signal amplification showed upregulation in the transcriptomic data in the resistant genotype (**Figure 5B**). The *MEKK3* gene is particularly responsible for activating the jasmonate signaling pathway (Taiz et al., 2015). Following the MAPK signaling, phytohormones, a chemical messenger, which transmits signals between the cells and initiates physiological responses, comes into play (Taiz et al., 2015). During aphid infestation, the sorghum resistant genotype an showed an elevated expression of JA and SA biosynthesis genes. Important JA (*LOX* and *OPR*) and SA (*PAL*) biosynthesis genes were highly upregulated in the resistant genotype. The *LOX* gene initiates the fatty acid oxidation pathways by breaking linolenic acid to produce oxylipins (death acids and green leaf volatiles) and JA, which have a defensive role in maize against aphids (Tzin et al., 2015) (**Figure 6A**). *SbLOX3* and *SbLOXo* also showed significant upregulation and their roles during sugarcane aphid infestation have been verified previously (Shrestha et al., 2021). Similarly, two *OPR* genes were upregulated more than five-fold in the resistant genotype. Stintzi et al. (2001) reported *OPR* in Arabidopsis to retain resistance to insect attack and fungal infection. Additionally, they have reported that oxylipin, *OPDA*, regulates gene expression in concert with JA to fine-tune the expression of defense genes.

SA, a product of the phenylpropanoid pathway, is an important signal involved in the activation of defense responses against biotic and abiotic stresses. The important genes in the phenylpropanoid pathway, *PAL*, *HCT*, *CAD*, and peroxidase, were all differentially expressed in the two genotypes during aphid infestation (**Figure 8A**). *PAL* catalyzes phenylalanine, the key step in this pathway that ultimately forms lignin, flavonoid and SA (**Figure 8A**) (Lv et al., 2017). The expression of *PAL* was significantly increased in corn seedlings and cotton infested by corn borer and cotton aphid, respectively (Lv et al., 2017). Similarly, *HCT* and *CAD* genes had elevated levels in the resistant genotype; these genes are important for lignin formation. Sugarcane aphid feeding on sorghum has shown an increase in the lignin content and the lignin pathway genes like *CAD* (Kundu et al., 2023). The RNAi-mediated suppression of *PAL* and *CAD* genes in wheat increased the penetration efficiency of *Blumeria graminis f.sp.tritici* (Bhuiyan et al., 2009). Lignin is a well-known defense polymer which forms a physical barrier to prevent the ingress or diffusion of toxins from pathogens (Sattler and Funnell-Harris, 2013).

The MAPK signaling, as well as phytohormones, activates an array of plant TFs that regulate several downstream genes during biotic stress (Kushalappa et al., 2016). The TFs, like MYB, WRKY, HD-Zip, and EREBP, were differentially expressed in sorghum genotypes (**Figures 5B**, **10C**). WRKY22 and WRKY33 genes were induced in both genotypes across all time points (**Figures 5B, C**). WRKY is the largest TF family in plants (Rushton et al., 2010) and has been found to regulate redox homeostasis, SA signaling, ET/JA-mediated cross communication, and camalexin biosynthesis, which are important against biotic stresses (Zheng et al., 2006; Birkenbihl et al., 2012). The overexpression of WRKY genes in *Oryza sativa* enhanced resistance to blast (*Magnaporthe grisea*) and leaf blight (*Xanthomonas oryzae*) through SA-mediated defense (Shimono et al., 2007). Next, the TFs of the MYB gene family one of the largest families and play an important role in activating hypersensitive cell death during pathogen attack and insect feeding through regulation of long-chain fatty acid synthesis (Ambawat et al., 2013). Four MYB TFs were highly upregulated in the resistant genotype (**Figure 10C**). A similar study has shown that the MYB TFs showed high upregulation and displayed circadian pattern in sorghum plants (Shrestha et al., 2024). A previous study in Arabidopsis reported MYB genes were associated with wound response or resistance to insects through a JA-dependent defense response (Cheong et al., 2002; Johnson and Dowd, 2004).The TFs of the HD-Zip gene family are unique to the plant kingdom and a recent study indicated the role of this gene family in regulation of ABA homeostasis and signaling as well as their potential role in plant protection from pathogen and abiotic stresses (Chew et al., 2013; Sessa et al., 2018).

Phytohormones SA and JA pathways and TFs lead to the activation of secondary metabolite–defense products in plants. We observed DEGs related to flavonoids, lignin, terpenoids, oxylipins, and glutathione. Two sorghum *CHS* genes related to the flavonoid pathway were upregulated more than four-fold in the resistance genotype according to the RNA-seq data (**Figure 9B**). The biological functions of flavonoids in plants are to provide defense against UV-B radiation, pathogen infection and insect infestation, nodulation, and pollen fertility (Falcone Ferreyra et al., 2012). Similarly, two GERD genes from the terpenoid pathway were also upregulated more than two-fold in the resistant genotype (**Figure 10A**). Arimura et al. (2004) reported that poplar tree infested with caterpillar (*Malacosoma disstria*) or mechanical wounding showed a strong increase of *GERD* and *LOX1* genes and an increase in release of (-)-germacrene, a sesquiterpene volatile. We also observed DEGs related to the linolenic acid pathway, which produce oxylipins (death acids and green leaf volatiles) and OPDA (**Figure 6**). Oxylipins, like death acids and green leaf volatiles, are known to have diverse functions in plant responses to infestation (Porta and Rocha-Sosa, 2002). Death acids like 10-oxo-11-phytoenoic acid (10-OPEA), 10-oxo-11-phytodienoicacid (10-OPDA), and 9-hydoxy-10E, 12Z-octadecadienoic acid (9-HOD) are derived from 9-LOXs, which suppressed the growth of fungi and insects (Christensen et al., 2015; Woldemariam et al., 2018). The OPDA can act independent of the JA pathway as the exogenous application of OPDA on maize JA-deficient plant showed enhanced resistance to corn leaf aphid (Varsani et al., 2019).

The activation of glutathione metabolism and expression of the enzyme in this pathway is correlated to the resistance to various biotic challenges and detoxification of ROS (Zechmann, 2014). The accumulation of ROS has been reported at the site of insect feeding and pathogen infection which caused cell death through a hypersensitive reponse, to prevent any further damage by pathogen or pest (Gechev et al., 2006). However, higher accumulations of ROS have a detrimental effect on plants; to cope with this, plants have developed antioxidant mechanisms to detoxify the ROS. Some of the major metabolites to detoxify ROS are glutathione, flavonoids, carotenoids, and ascorbic acids (Ahmad et al., 2009). Most resistant genotypes exhibit antioxidant gene upregulation during stress. Our study showed 15 *GST* genes, a regulator of the glutathione pathway, upregulated in the resistant genotype. A previous study reported that *GST1* (*Sobic.001G318200*) and *GST3* (*Sobic.001G319500*) were highly upregulated (4- to 81-fold) in the resistant genotype of sorghum (Tx2783) during sugarcane aphid infestation (Pant and Huang, 2022). Another gene, peroxidase, which was also upregulated in the resistant genotype during aphid infestation, has multiple functions. Six peroxidase genes were upregulated in the resistant genotype (**Figure 10B**). *Peroxidase* genes code for ROS-detoxifying enzymes, are involved in oxidative signal transduction, regulate the redox and Ca^2+^ homeostasis, and activate defense genes (Kawano, 2003; Gulsen et al., 2010).

**Figure 12:**
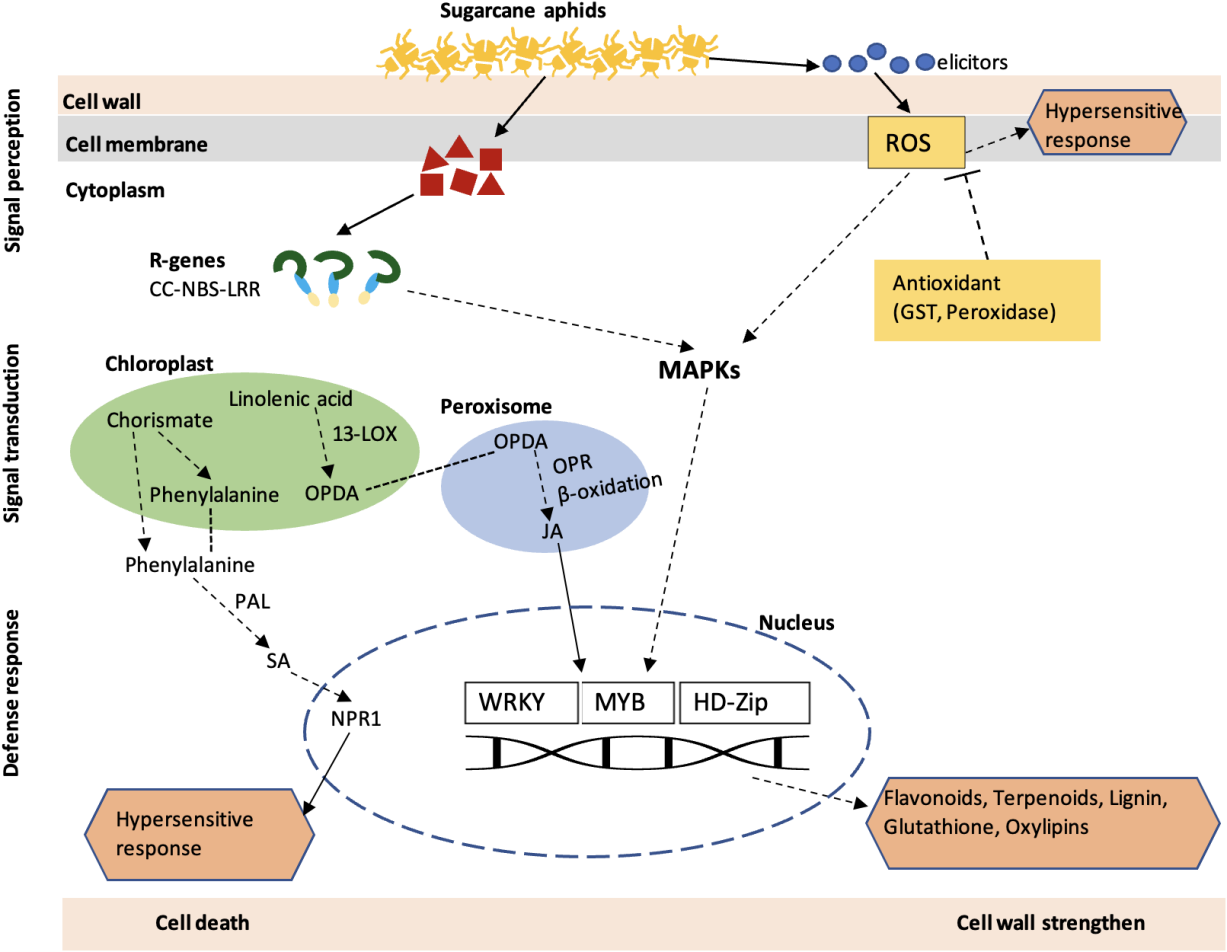
A schematic overview of the molecular responses of sorghum plant to sugarcane aphid infestation. Upon wounding by aphids, the host plants initiate a series of events, which includes signal perception (R genes), signal transduction (MAPK signaling and phytohormones) and defense response (TFs, flavonoids, and terpenoids). CC-NLR, coiled-coil nucleotide-binding leucine-rich repeat proteins; *ROS*, *Reactive oxygen species*; *MAPK*, *Mitogenactivated protein*; *PAL*, *phenylalanine ammonialyase*; SA, Salicylic acid; JA, Jasmonic acid; *LOX*, *lipoxygenase*; WRKY, WRKY transcription factor; MYB, transcription factor: *GST*, *glutathione Stransferase*; HD-ZIP, homeobox leucine zipper protein; *OPDA*, *oxo-phytodienoic acid*; *OPR*, *12-oxophytodienoic acid reductase*.

The phytohormone treatment indicates the role of JA and SA in sugarcane aphid resistance in sorghum. The SA- and JA-treated susceptible genotype showed a significant decrease in aphid number, plant mortality, and damage ratings (**Figure 11**). SA generally induces plant defense against biotrophic pathogens (Vos et al., 2015). A previous study showed that *Brassica napus* treated with SA decreased the *Brevicoryne brassicae* population through antibiosis mechanism (Khoshfarman-Borji et al., 2020). Similarly, exogenous application of SA enhanced the resistance of *Oryza sativa* to the *Nilparvata lugens* (Guo-Zhang et al., 2003). The activation of the SA pathway in resistant plant is pointed out to be a general mechanism of antibiosis or antixenosis (Morkunas et al., 2011). The exogenous application of JA in soybean plants reduced the soybean aphid population (Yates-Stewart et al., 2020), soybean thrips, and soybean aphids (Selig et al., 2016). Previously, we have shown that resistant sorghum lines infested with sugarcane aphid revealed significantly higher amounts of SA and JA during phytohormone profiling through liquid chromatography–mass spectrometry (Huang et al., 2022). Evidently, our results obtained from SA- and JA-treated sorghum plants are consistent with those reports in other plant species; therefore, our result suggests that SA and JA can induce the resistance in sorghum against aphids and further suggests that antibiosis mechanisms of resistance are involved. Overall, our result from this study suggests a series of genes and biosynthesis pathways are involved in host-aphid interaction, summarized in **Figure 12**. These series of genes ultimately form defense compounds like flavonoids, terpenoids, and oxylipins which have a direct effect on aphids by influencing their behavior, growth, and development (Blée, 2002; Simmonds, 2003). In addition, lignin production strengthens the cell wall and limits penetration by invasive pests or pathogens. Plant HR activated through ROS and SA pathways, leading to cell death and avoiding any further damage.

## 5 Conclusion

In summary, transcriptomic analysis demonstrated differential expression of a series of genes in the resistant Tx2783 and susceptible BTx623 sorghum genotypes in response to infestation by sugarcane aphid. The host plant infested with aphids initiated a large transcriptional reprogramming and subsequent molecular events starting from the early time point (3 dpi) in the resistant genotype. Such altered transcriptional activities of defense-related genes included signal perception (*NLR* genes), signal transduction [MAPK signaling and phytohormones (SA and JA)], and defense responses (TFs, flavonoids, and terpenoids). These defense-related genes and pathways are the underlying mechanisms of the resistant plants to defend themselves against aphid attack. Future research should focus on quantifying metabolites like oxylipins, lignin, and terpenoids in sorghum resistance to aphids. Similarly, molecular experiments to analyze the function of those candidate genes identified in this study will further validate their roles in plant-aphid interactions, leading to a successful self-protection of the host plant.

## Data availability statement

The datasets presented in this study can be found in online repositories. The names of the repository/repositories and accession number(s) can be found below: BioProject, PRJNA961311.

## Author contributions

KS: Investigation, Writing – review & editing, Data curation, Formal analysis, Software, Validation, Visualization, Writing – original draft. JH: Data curation, Formal analysis, Investigation, Visualization, Writing – review & editing, Conceptualization. LY: Investigation, Writing – review & editing, Methodology. AD: Investigation, Methodology, Writing – review & editing, Resources. YH: Investigation, Resources, Writing – review & editing, Conceptualization, Funding acquisition, Project administration, Supervision.

## Supplementary material

Supplementary Table 1 Primers used for the RT-qPCR.

Supplementary Table 2 Summary of RNA-seq reads and mapping.

Supplementary Table 3 Number of DEGs in sorghum genotypes (Tx2783 and BTx623) in response to sugarcane aphid feeding at four different time-points.

Supplementary Table 4 Number of shared and unique DEGs in sorghum genotypes (Tx2783 and BTx623) in response to sugarcane aphid attack at four different time-points.

Supplementary Table 5 Number of unique DEGs in resistant Tx2783 and susceptible BTx623 genotypes from all four time-points in each sorghum genotype along with the number of GO terms annotated.

Supplementary Table 6 Number of KEGG terms annotated from the DEGs of resistant Tx2783 and susceptible BTx623 sorghum genotypes exposed to sugarcane aphid infestation.

Supplementary Table 7 GO terms annotation from the DEGs of resistant Tx2783 and susceptible BTx623 sorghum genotypes exposed to sugarcane aphid infestation (Excel sheet).

Supplementary Table 8 KEGG annotation from the DEGs of resistant Tx2783 and susceptible BTx623 sorghum genotypes exposed to sugarcane aphid infestation (Excel sheet).

Supplementary Figure 1 Sorghum genotypes infested with sugarcane aphid at different time points.

Supplementary Figure 2 RNA-seq analysis workflow (green boxes) and the visualization (light orange boxes). The italicized letters in the boxes are the packages or tools used for the RNA-seq analysis. The blue text represents the file type or output from earlier step.

Supplementary Figure 3 The scatterplot showing the correlation between RT-qPCR and RNA-seq data of the selected differentially expressed genes.
