# Supplementary material for: Integrated transcriptomic and pathway analyses of sorghum plants revealed the molecular mechanisms of host defense against aphids
Source: Front Plant Sci. 2024 Jun 6;15:1324085. doi: 10.3389/fpls.2024.1324085 (PMC11187118; doi:10.3389/fpls.2024.1324085)
Supplement: Supplementary Table 1 — Primers used for the RT-qPCR. [file Table_1.docx]

**Table S1.** Primers used for the RT-PCR.

| S.No. | Gene symbol | Gene ID | Forward primer | Reverse primer |
| --- | --- | --- | --- | --- |
|  | *OPR* | Sobic.010G084600 | CCCTTTGCCGACTACTTTGA | CATTCGAGGTTCCACCATGT |
|  | *OPR* | Sobic.010G084700 | CCTTTGCCGACTACTTTGATTG | CCATGTGGCAGTAGAGGAAA |
|  | *LOX3* | Sobic.003G385500 | AGTTCTTCCTCAAGACCATCAC | AGAAGAAGACGCGGTTGTAG |
|  | *LOXo* | Sobic.001G125700 | GAGGCTGTTCTTCCGTTGATA | CTTCCGGACCAAGTTCATAGAG |
|  | *HCT* | Sobic.010G066601 | GCTCATGTACTACAGTGGGTTC | GCGAACACCTTCCTGAACT |
|  | *HCT* | Sobic.007G142100 | ATGTTCGTGCCGTTCGT | ATGGTCACGTGGTGGATG |
|  | *PAL* | Sobic.004G220600 | CATGAAGCTTGCCAAGAAGC | GGCACGGATAACCTCAATCT |
|  | *PAL* | Sobic.004G220700 | CATGAAGCTTGCCAAGAAGC | CGCACGGATAACCTCAATCT |
|  | *CAD* | Sobic.006G014700 | GAAGCATTCCTTGGGACCTATC | CAAAGATGAGCGGGAACGA |
|  | *Peroxidase* | Sobic.009G144600 | CTAGAGGCGCATTGGAGAAC | GAAGGGTCCGACAGCATAAC |
|  | *CHS* | Sobic.005G136300 | AGATCACTGCCGTCACATTC | GGGTCAGCACCGACAATAA |
|  | *ANR* | Sobic.006G227000 | CTGCTAGAAGAAGGCAGCTT | CAGGCTCGATCAGTTCTTTCT |
|  | *FLS* | Sobic.004G310100 | CCTGCACAAGGTCAACTTCTA | TCATTGGGCACGAGGATG |
|  | *IAA* | Sobic.009G203700 | TGTCAGCTCATCTAAGCAATCC | GGGATGCCGTCCATGTTTAT |
|  | *GH3* | Sobic.003G306500 | CTACTACAAGAGCGACCACTTC | GCACAGCATCTGAGAGTACAT |
|  | *SAUR* | Sobic.001G146500 | CGACGAGGAGGAGAAGGT | ACGGAAGGGTCGCTGAG |
|  | *TGA* | Sobic.002G247300 | CACTGGCCAAGAAGGATCATAG | GACTCCAGTTGCTGGATGTAAG |
|  | *JAZ9* | Sobic.001G482700 | TCAAGAGGTTCCTCGAGAAGAG | TCCTTAACAGCAGGAGGCT |
|  | *JAZ16* | Sobic.006G056400 | GCCACTCACAAGAACCAAATC | GTTCCTCCCGATCCCATTATTC |
|  | *RbOHD* | Sobic.005G139700 | GAGGAGGAGGTCAAAGAGATCA | GGGTCTAGCTCCTCCATGATTA |
|  | *MAPK-17/18* | Sobic.003G268700 | GATTTCTTGGCCTGCTGTTTC | TGTCGTCATGATCCGCAAAT |
|  | *MAPK-17/18* | Sobic.009G217500 | TCGGAGACGACAAGCTATGA | TCATCGCCAACATCCAAGTC |
|  | *WRKY33* | Sobic.003G341100 | GACTTCTCGTTCCAGACAGC | TGCTGCTGCTGACTTCTATAC |
|  | *WRKY22* | Sobic.003G226600 | CAAGAAGAGCCAGCTGAAGA | ATAAGGCGAGCCCTTGATG |
|  | *MEKK3* | Sobic.004G176900 | GCAGGAAGTAGACATGCTTAGAC | GGACTGAACCTCCAGAAACATAC |
|  | *WRKY33* | Sobic.009G171600 | CAGTACACCGACTTCACGTTC | CAGCACTGATGACTGCAGATAG |
|  | *CHS* | Sobic.007G170400 | AAATCACCCTCGTCTGCTTC | CGGAAACCATCTCGAACACT |
|  | *GERD* | Sobic.001G173000 | TAACGCACGGAGAGGTAGAA | CGGGTTACTTGACCAGCTAATG |
|  | *GERD* | Sobic.009G009300 | GTGGACAAACTGAGGGAAGAAG | CGAACAGGTGATCCAAGCATAG |
|  | *MYB* | Sobic.002G423300 | CACCGACAACGAGATCAAGAA | CATCCAGATGTAGCGCATGA |
|  | *MYB* | Sobic.001G397900 | CAACATCTCCAAGGAGGAAGAG | CGAGTTCCAGTGGTTCTTGAT |
|  | *α-Tub* | Sobic.001G107200 | GAGGTGACGATGCTTTCAACAC | CACAGGTCAACAATCTCCTTGC |

**Table S2.** Summary of RNA-seq reads and mapping.

| Sample name | Tx2783 | | | | | BTx623 | | | | | | Average |
| --- | --- | --- | --- | --- | --- | --- | --- | --- | --- | --- | --- | --- |
|  | 0dpi | 3dpi | 6dpi | 9dpi | 12dpi | 0dpi | 3dpi | 6dpi | 9dpi | 12dpi |  | |
| Total reads | 56622218 | 57620070 | 55251084 | 56911444 | 55964864 | 52141630 | 39099780 | 53688578 | 59613386 | 46039992 | 53.3 M | |
| Total mapped reads | 52352270 | 53258050 | 52067006 | 53285437 | 52278041 | 49837231 | 37864926 | 49709879 | 48982524 | 43196612 | 49.3 M | |
| Uniquely mapped reads | 50382211 | 51482722 | 50617791 | 51769294 | 50650527 | 48304747 | 36937827 | 47933545 | 47447278 | 42396102 | 47.8 M | |
| Multiple mapped reads | 1970059 | 1775328 | 1449215 | 1516143 | 1627514 | 1532484 | 927099 | 1776334 | 1535246 | 800510 | 1.49 M | |
| Total mapping rate | 92.46% | 92.43% | 94.24% | 93.63% | 93.41% | 95.58% | 96.84% | 92.59% | 82.17% | 93.82% | 92.61% | |
| Uniquely mapping rate | 88.98% | 89.35% | 91.61% | 90.96% | 90.50% | 92.64% | 94.47% | 89.28% | 79.59% | 92.09% | 89.80% | |
| Multiple mapping rate | 3.48% | 3.08% | 2.62% | 2.66% | 2.91% | 2.94% | 2.37% | 3.31% | 2.58% | 1.74% | 2.81% | |

**Table S3.** Number of DEGs in sorghum genotypes (Tx2783 and BTx623) in response to sugarcane aphid herbivory at four different time-points.

| Genotype | Time points | Upregulated | Downregulated | Total |
| --- | --- | --- | --- | --- |
| Tx2783 | 3dpi | 1196 (53.94%) | 1021 (46.05%) | 2217 |
|  | 6dpi | 1469 (41.57%) | 2064 (58.42%) | 3533 |
|  | 9dpi | 1535 (43.95%) | 1957 (56.04%) | 3492 |
|  | 12dpi | 1958 (67.63%) | 937 (32.36%) | 2895 |
| BTx623 | 3dpi | 2115 (42.34%) | 2880 (57.65%) | 4995 |
|  | 6dpi | 1896 (61.55%) | 1184 (38.44%) | 3080 |
|  | 9dpi | 2232 (33.08%) | 4515 (66.91%) | 6747 |
|  | 12dpi | 2352 (41.46%) | 3320 (58.53%) | 5672 |

**Table S4.** Number of shared and unique DEGs in sorghum genotypes (Tx2783 and BTx623) in response to sugarcane aphid herbivory at four different time-points.

| Time-points | DEGs | Unique DEGs | | Shared DEGs |
| --- | --- | --- | --- | --- |
|  |  | Tx2783 | BTx623 |  |
| Early (3 and 6 dpi) | Up | 1445 | 2215 | 553 |
|  | Down | 1256 | 2228 | 1100 |
| Late (9 and 12 dpi) | Up | 1494 | 2242 | 886 |
|  | Down | 733 | 3546 | 1713 |

**Table S5.** Number of unique DEGs in resistant Tx2783 and susceptible BTx623 genotype from all four time-points in each sorghum genotype along with number of GO terms annotated.

| Genotypes | Number of unique DEGs in all 4 time-points | GO terms annotated |
| --- | --- | --- |
| Tx2783_upreg | 2295 | 246 |
| Tx2783_down | 3252 | 500 |
| BTx623_upreg | 3862 | 309 |
| BTx623_down | 6081 | 552 |

**Table S6.** Number of KEGG terms annotated from the DEGs of resistant Tx2783 and susceptible BTx623 sorghum genotypes exposed to sugarcane aphid herbivory.

| Genotypes | Days post infestation (dpi) | KEGG terms annotated | | |
| --- | --- | --- | --- | --- |
|  |  | Upregulated (pathways #) | Downregulated  (pathways #) | Total  (pathways #) |
| Tx2783 | 3dpi | 347 (90) | 319 (83) | 666 (173) |
|  | 6dpi | 394 (98) | 690 (125) | 1084 (223) |
|  | 9dpi | 534 (131) | 798 (148) | 1332 (279) |
|  | 12dpi | 677 (139) | 385 (107) | 1062 (246) |
| BTx623 | 3dpi | 597 (120) | 931 (144) | 1528 (264) |
|  | 6dpi | 437 (106) | 404 (103) | 841 (209) |
|  | 9dpi | 838 (159) | 1830 (183) | 2668 (342) |
|  | 12dpi | 908 (111) | 1349 (174) | 2257 (285) |
