# Supplementary figures and images for: Integrated transcriptomic and pathway analyses of sorghum plants revealed the molecular mechanisms of host defense against aphids
Source: Front Plant Sci. 2024 Jun 6;15:1324085. doi: 10.3389/fpls.2024.1324085 (PMC11187118; doi:10.3389/fpls.2024.1324085)

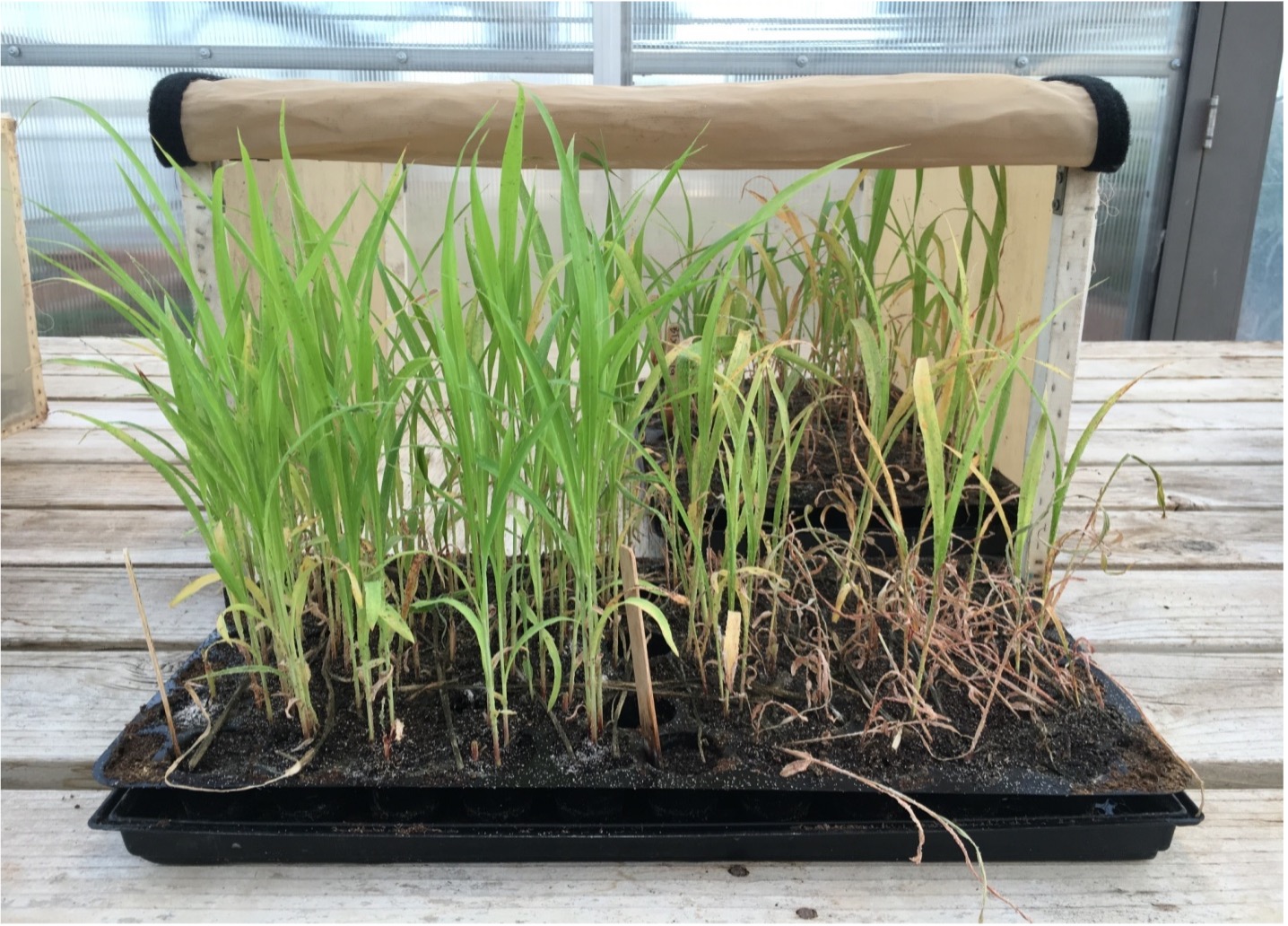

Supplement: Supplementary Figure 1 — Sorghum genotypes infested with sugarcane aphid at different time points. [file Image_1.jpg]

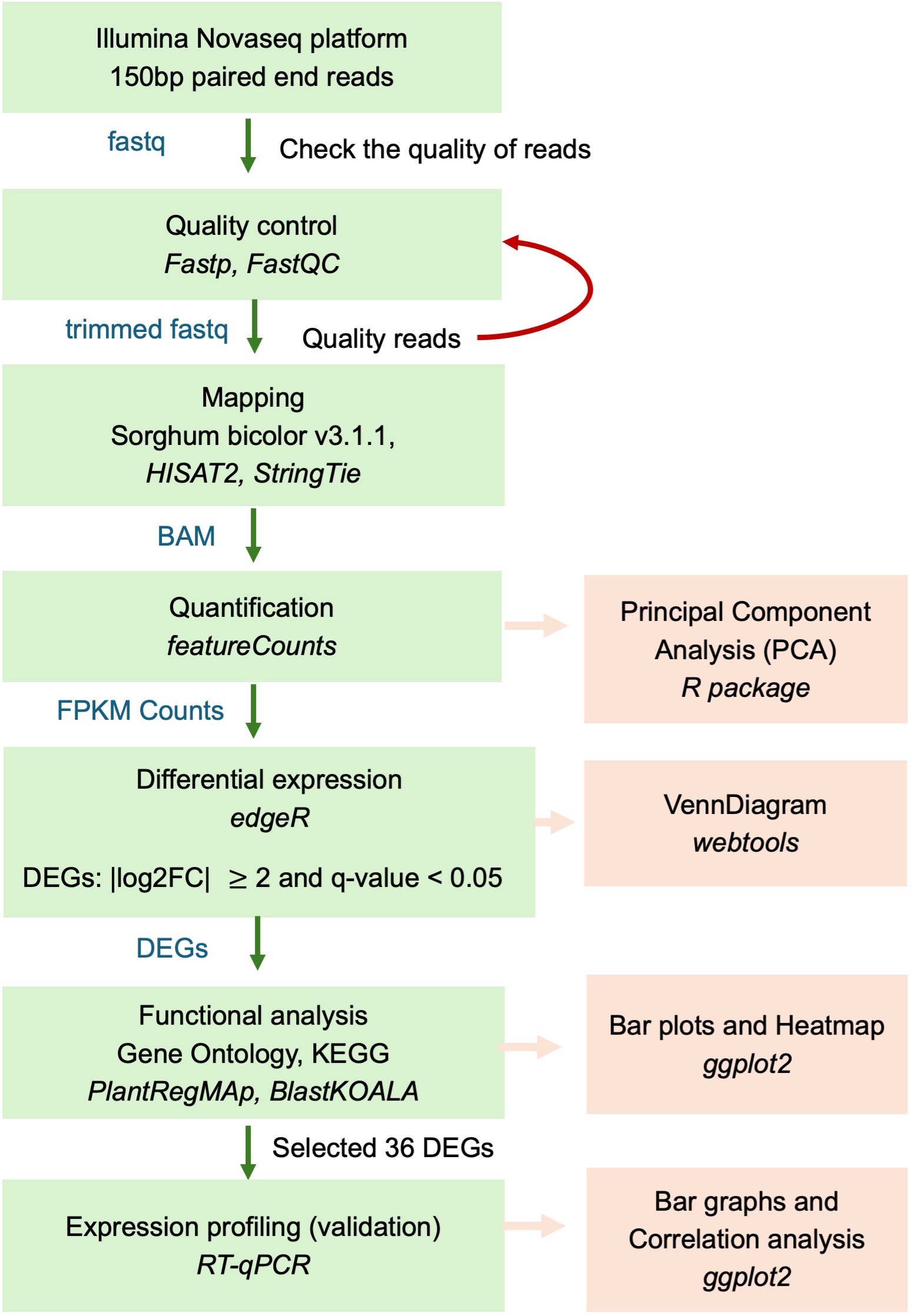

Supplement: Supplementary Figure 2 — RNA-seq analysis workflow (green boxes) and the visualization (light orange boxes). The italicized letters in the boxes are the packages or tools used for the RNA-seq analysis. The blue text represents the file type or output from earlier step. [file Image_2.jpg]

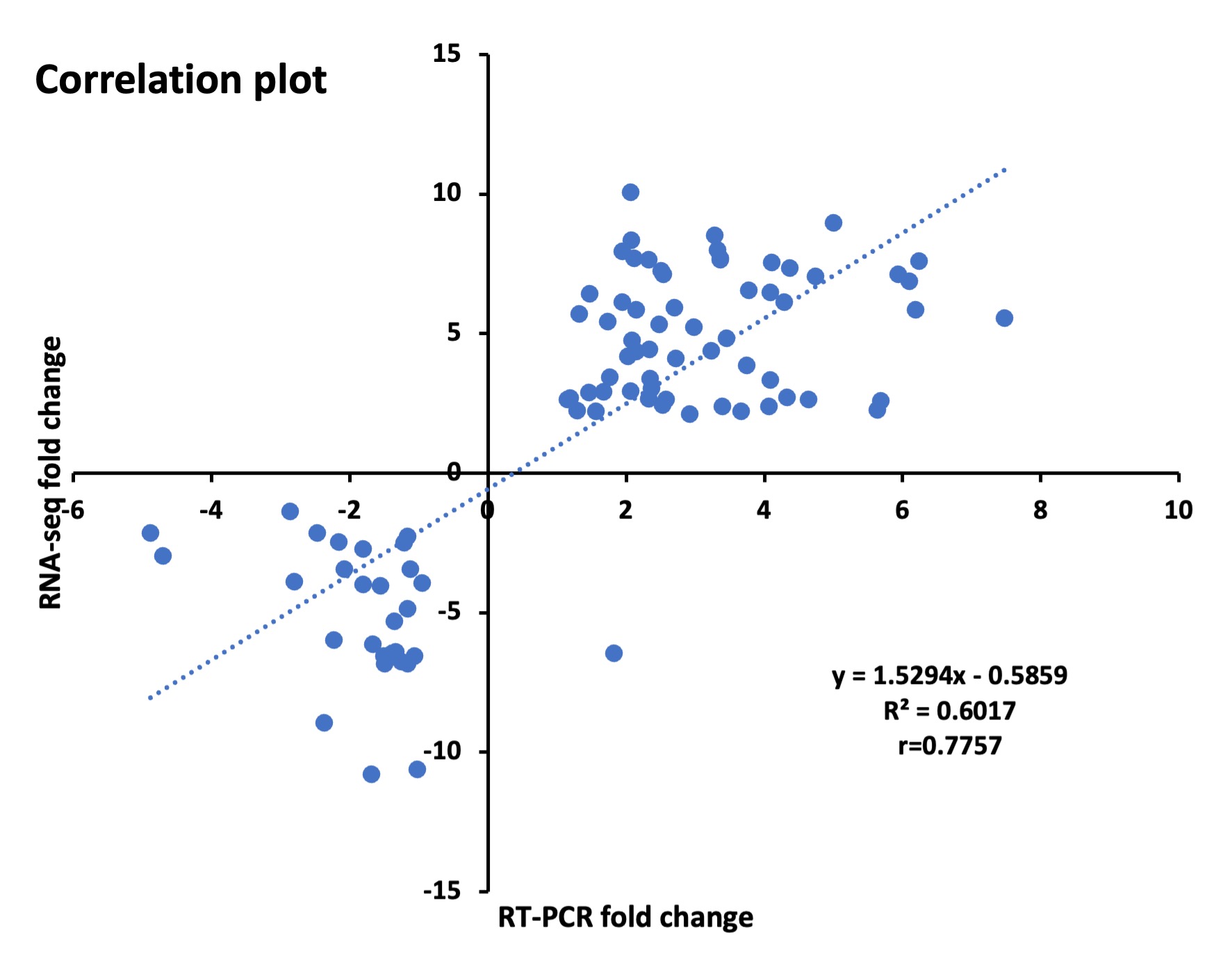

Supplement: Supplementary Figure 3 — The scatterplot showing the correlation between RT-qPCR and RNA-seq data of the selected differentially expressed genes. [file Image_3.jpg]
